# Electrical control of induced magnetism in an air-stable two-dimensional semiconductor

**DOI:** 10.1126/sciadv.aeb0659

**Published:** 2026-09-02

**Authors:** Qi Zhang, Mithun Ghosh, Yaroslav Zhumagulov, Oldřich Cicvárek, Yuan Chen, Fangchao Long, Yijie Lin, Ali Al Mejamai, Johan Félisaz, Iva Plutnarová, Jianmin Yang, Hue Thi Bich Do, Jan Plutnar, Michel Bosman, Shengqiang Zhou, Goki Eda, Oleg V. Yazyev, Zdeněk Sofer, Ahmet Avsar

**Affiliations:** ^1^ Department of Materials Science and Engineering, National University of Singapore, Singapore 117575, Singapore.; ^2^ Institute of Physics, Ecole Polytechnique Fédérale de Lausanne (EPFL), CH-1015 Lausanne, Switzerland.; ^3^ Department of Inorganic Chemistry, University of Chemistry and Technology Prague, Technická 5, 166 28 Prague 6, Czech Republic.; ^4^ Department of Physics, National University of Singapore, Singapore 117542, Singapore.; ^5^ Helmholtz-Zentrum Dresden-Rossendorf, Institute of Ion Beam Physics and Materials Research, Bautzner Landstraße 400, D-01328 Dresden, Germany.; ^6^ Department of Chemistry, National University of Singapore, Singapore 117543, Singapore.; ^7^ Centre for Advanced 2D Materials, National University of Singapore, Singapore 117546, Singapore.

## Abstract

Dilute magnetic semiconductors (DMSs) provide a platform for electrically controlling spin interactions; however, conventional systems face limited gate tunability and structural disorder. Here, we demonstrate gate-switchable magnetism in air-stable, dilute Fe-doped PtSe_2_, a two-dimensional (2D) DMS that remains structurally homogeneous down to the atomically thin limit. Bulk crystals exhibit ferromagnetism with a Curie temperature of 320 kelvin, and this coupling persists in metallic devices down to seven layers. As thickness and carrier concentration further decrease, the system transitions to antiferromagnetic order, with a gate-tunable Néel temperature reaching 105 kelvin in five-layer semiconducting devices. Our first-principles calculations reveal a carrier-density–dependent crossover from Ruderman-Kittel-Kasuya-Yosida–mediated ferromagnetism to superexchange-driven antiferromagnetism. These findings demonstrate how induced magnetic order evolves from bulk to the 2D limit, providing a pathway to functional spintronic devices with electrically controlled magnetic states.

## INTRODUCTION

The interplay between charge carriers, dimensionality, and magnetism is a central theme in condensed matter physics, with implications for the development of quantum materials, spin-based logic, and next-generation memory technologies (*1*–*3*). Among different magnetic orders, antiferromagnetism is a compelling platform for spintronics, offering immunity to stray magnetic fields, ultrafast spin dynamics, and compatibility with dense, energy-efficient device architectures (*4*, *5*). The ability to electrically modulate antiferromagnetic (AFM) order, particularly in two-dimensional (2D) materials, would represent an important step toward reconfigurable spintronic functionality (*6*, *7*). While intrinsic 2D antiferromagnets such as MnPS_3_, CrI_3_, and CrSBr exhibit long-range magnetic ordering (*8*) with electric-field (*7*, *9*–*11*) and intercalation-induced tunability (*12*–*14*), their environmental instability remains a limitation for their technological applicability (*15*). An alternative route is provided by dilute magnetic semiconductors (DMSs), where magnetic dopants introduce localized moments into nonmagnetic hosts, enabling carrier-mediated control of magnetic interactions (*16*, *17*). In well-studied systems like GaMnAs, ferromagnetic (FM) ordering arises through hole-mediated exchange and can be modulated by gating (*17*–*19*). Translating this concept to 2D materials offers a promising strategy for realizing tunable magnetism (*20*), yet the stabilization of AFM phases within a truly 2D DMS framework remains unexplored.

In such atomically thin systems, the dominant magnetic exchange mechanism is expected to vary sensitively with carrier concentration (*1*, *21*, *22*). In the metallic regime, itinerant electrons mediate long-range Ruderman-Kittel-Kasuya-Yosida (RKKY) interactions that often favor oscillatory and FM coupling (*17*). Conversely, in semiconducting or insulating states where electronic screening is suppressed, localized spins can couple via superexchange, a mechanism typically mediated through anion orbitals, which typically favors AFM alignment and becomes stronger as carrier concentration decreases (*23*). This contrast presents a unique opportunity: By engineering a carrier-tunable 2D DMS, one may stabilize different magnetic ground states and drive transitions between them through electrostatic gating or structural control. Understanding and leveraging this competition is essential for designing magnetic systems with electrically switchable phases.

Layered transition metal dichalcogenides represent an ideal platform for such studies (*20*), owing to their air stability, tunable electronic structures, and compatibility with electrostatic gating (*24*). Among these, PtSe_2_ is particularly attractive due to its strong thickness-dependent band structure: While bulk PtSe_2_ is metallic, reducing its thickness below six layers induces a semiconductor transition via bandgap opening (*25*–*32*). It is also air stable (*29*, *32*, *33*). This enables access to both semiconducting and metallic regimes within the same material system. Additionally, layer-dependent magnetization in both metallic (*33*) and semiconducting (*34*) PtSe_2_ has been experimentally observed to arise from intrinsic Pt vacancies, in agreement with theoretical predictions (*35*, *36*), with magnetic ordering observed up to 6 K. For DMS structures, first-principles calculations predict that, while substitution of Pt with Fe or Mn stabilizes AFM order, even in the presence of Pt vacancies, doping with elements such as V, Os, or Ru induces FM coupling with expected transition temperatures up to 380 K, highlighting PtSe_2_ as a highly versatile platform for engineering induced magnetic order through selective doping (*37*, *38*). Realizing this behavior, however, requires suppression of dopant clustering and electronic disorder; challenges that are particularly amenable to study in 2D materials, where surface-sensitive techniques like scanning transmission electron microscopy (STEM) enable direct verification of dopant homogeneity and substitution (*39*).

Here, we investigate ∼5.5% Fe-doped PtSe_2_ using magnetotransport measurements across a range of thicknesses, carrier densities, and temperatures. In five- (5L) and six-layer (6L) devices, corresponding to the semiconducting and semiconducting to metal transition regimes, we observe robust AFM ordering, with a Néel temperature (*T*_N_) that increases monotonically as carrier concentration is reduced, reaching up to 55 and 105 K in 6L and 5L devices, respectively. The magnetic response is highly sensitive to electrostatic gating, allowing magnetic hysteresis to be switched on and off. As thickness and carrier concentration increase, AFM interactions weaken and the system undergoes a transition to FM ordering: Thick metallic devices exhibit FM signatures in transport, and bulk samples display robust FM behavior with a Curie temperature (*T*_C_) exceeding room temperature (RT). This evolution is corroborated by our density functional theory (DFT) calculations, which reveal a transition from superexchange-driven AFM to RKKY-mediated FM coupling as carrier concentration increases. These findings demonstrate that the Fe-PtSe_2_ system provides a robust, air-stable platform for probing the interplay between magnetism and carrier concentration in two dimensions without compromising its intrinsic electronic properties and establish a foundation for gate-controllable spintronic devices based on 2D antiferromagnetism.

## RESULTS

### Basic characterisation of dilute Fe-doped PtSe_2_

**Fig. 1.**
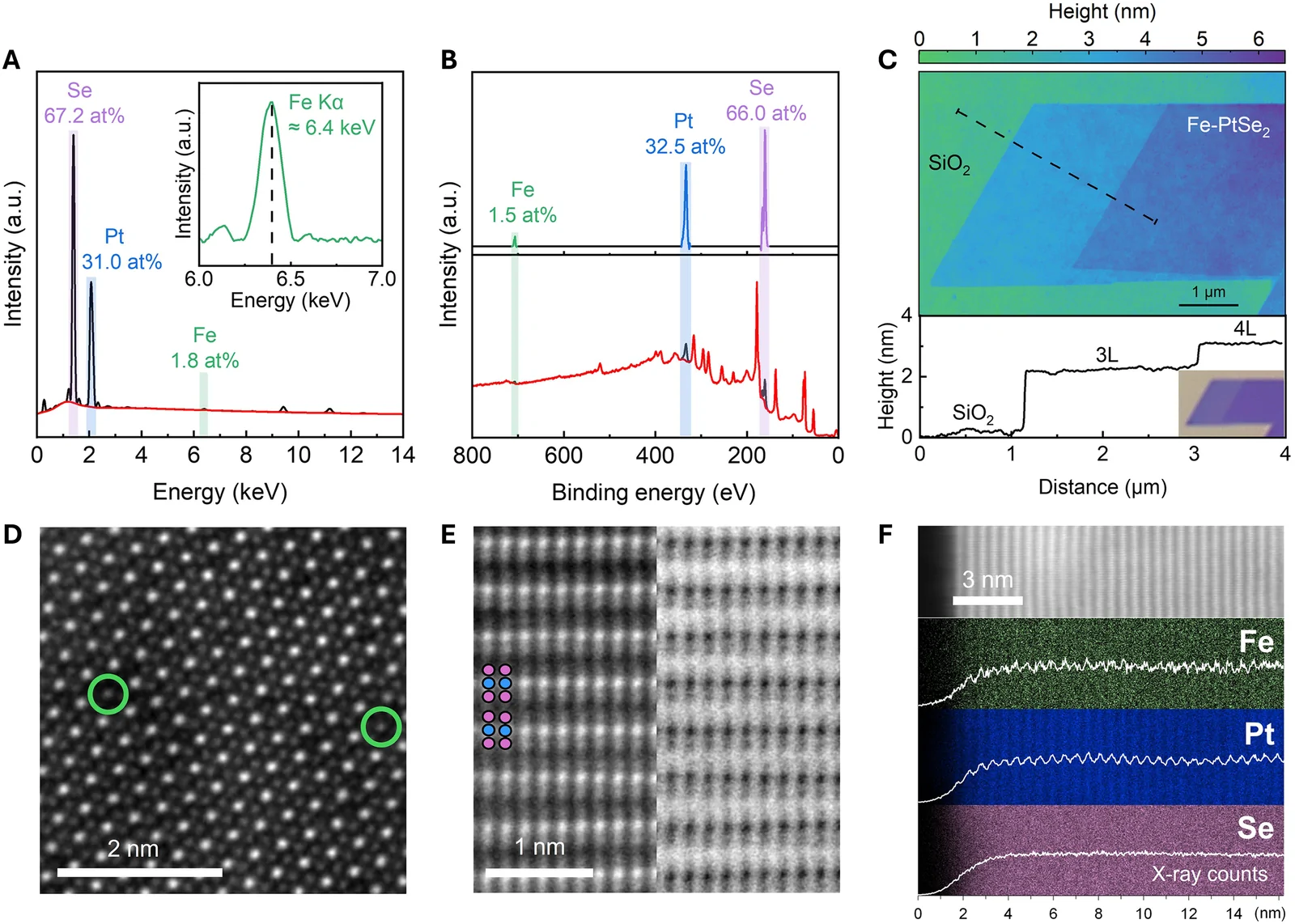
Material characterization. (**A**) Energy-dispersive x-ray spectroscopy (EDS) spectrum of bulk Fe-doped PtSe_2_ sample with the highlighted areas of Fe Kα (green), Pt Mα (blue), and Se Lα (purple) electrons used for determination of the elemental composition. The red line is the background correction, and the black line is the actual spectrum. Inset: Highlighted Fe Kα characteristic peak. (**B**) X-ray photoelectron spectroscopy (XPS) spectrum of bulk Fe-doped PtSe_2_ sample with the highlighted areas of Fe 2p_3/2_ (green), Pt 4d_3/2_ (blue), and Se_3p_ (purple) electrons used for determination of the elemental composition. In the bottom panel, the red line is the background correction, and the black line is the actual spectrum. Top panel shows the background-subtracted spectrum. (**C**) Top panel: Atomic force microscopy image of Fe-doped PtSe_2_ flake with varying thickness from 3L to 4L. Bottom: Height measurement for the corresponding dashed line in the top panel. Inset: Optical image of Fe-doped PtSe_2_ flake in the top panel. (**D**) High-resolution angle annular dark-field (HAADF) STEM image of a bilayer Fe-doped PtSe_2_ flake, with green circles representing substitutional Fe atoms. (**E**) Cross-sectional HAADF (left)/annular bright-field (right) STEM images of Fe-doped PtSe_2_. The blue circles indicate the location of the Pt columns; the purple circles represent the Se columns. (**F**) HAADF image (grayscale) and STEM-EDS elemental maps (colored) of Fe-doped PtSe_2_. The low Fe concentration results in a weaker EDS signal; however, the integrated elemental profile shows that Fe (green) is located in the same planes as Pt (blue), indicating substitutional replacement of Pt by Fe.

Fe-doped PtSe_2_ single crystals were synthesized using a chemical vapor transport method adapted from our previously established protocol (see Materials and Methods). We explored a range of Fe concentrations to improve doping homogeneity and structural quality; here, we present data from a sample targeting a nominal 10% substitution of Pt by Fe. For bulk crystals, the Fe concentrations revealed by energy-dispersive x-ray spectroscopy (EDS) [1.8 atomic % (at %) Fe, corresponding to ∼5.5% Fe doping] (Fig. 1A) and x-ray photoelectron spectroscopy (XPS) (1.5 at % Fe, corresponding to ∼4.4% Fe doping) (Fig. 1B) are consistent, reflecting a macroscopic or surface-averaged elemental composition. The lower Fe concentration compared with the nominal 10% is typical for substitutional doping as the limited solubility of magnetic elements restricts the achievable doping level (*32*, *40*).

Upon thinning the bulk crystal into the 2D regime, Fe-doped PtSe_2_ retains its atomically flat, layered structure with clean terraces and step heights of ∼0.65 nm (Fig. 1C), consistent with the expected interlayer spacing (*34*). Complementary EDS measurements performed on exfoliated flakes (fig. S1) show similar Fe concentrations to those of the bulk crystals. The material remains stable under ambient conditions and even after 250°C annealing, confirming the robustness of Fe-doped PtSe_2_ down to the few-layer limit (fig. S2).

To further investigate the doping strategy and crystal quality at the atomic level, high-resolution angle annular dark-field (HAADF) STEM was used to image a bilayer sample in a top-view geometry (Fig. 1D), which reveals a well-defined atomic arrangement and high crystallinity. Several sites of Pt columns display lower intensities, and two of such examples are highlighted with green circles, which represent the substitutional Fe atoms. No lattice relaxation was found in intensity profiles taken from three directions across the circled position, further supporting this assignment (fig. S3). By contrast, if transition-metal atoms were intercalated between the pristine PtSe_2_ layers, then an increased column intensity would be expected (*41*). In addition, a cross-sectional HAADF-STEM imaging of a ∼20-nm-thick sample (Fig. 1E, left) reveals a layered structure without atoms intercalated in between the layers, further confirming substitutional Fe doping. The corresponding annular bright-field STEM image (Fig. 1E, right) shows uniform dark contrast across all atomic columns. Both scanning electron microscopy–EDS and STEM-EDS elemental mapping of bulk and thin crystals (figs. S4 and S5 and Fig. 1F) further confirm homogeneous atomic distribution across the samples, with no evidence of phase segregation or clustering of substituted elements, reinforcing the successful substitution of Fe atoms into pristine PtSe_2_.

### Up to RT ferromagnetism in thick metallic regime

**Fig. 2.**
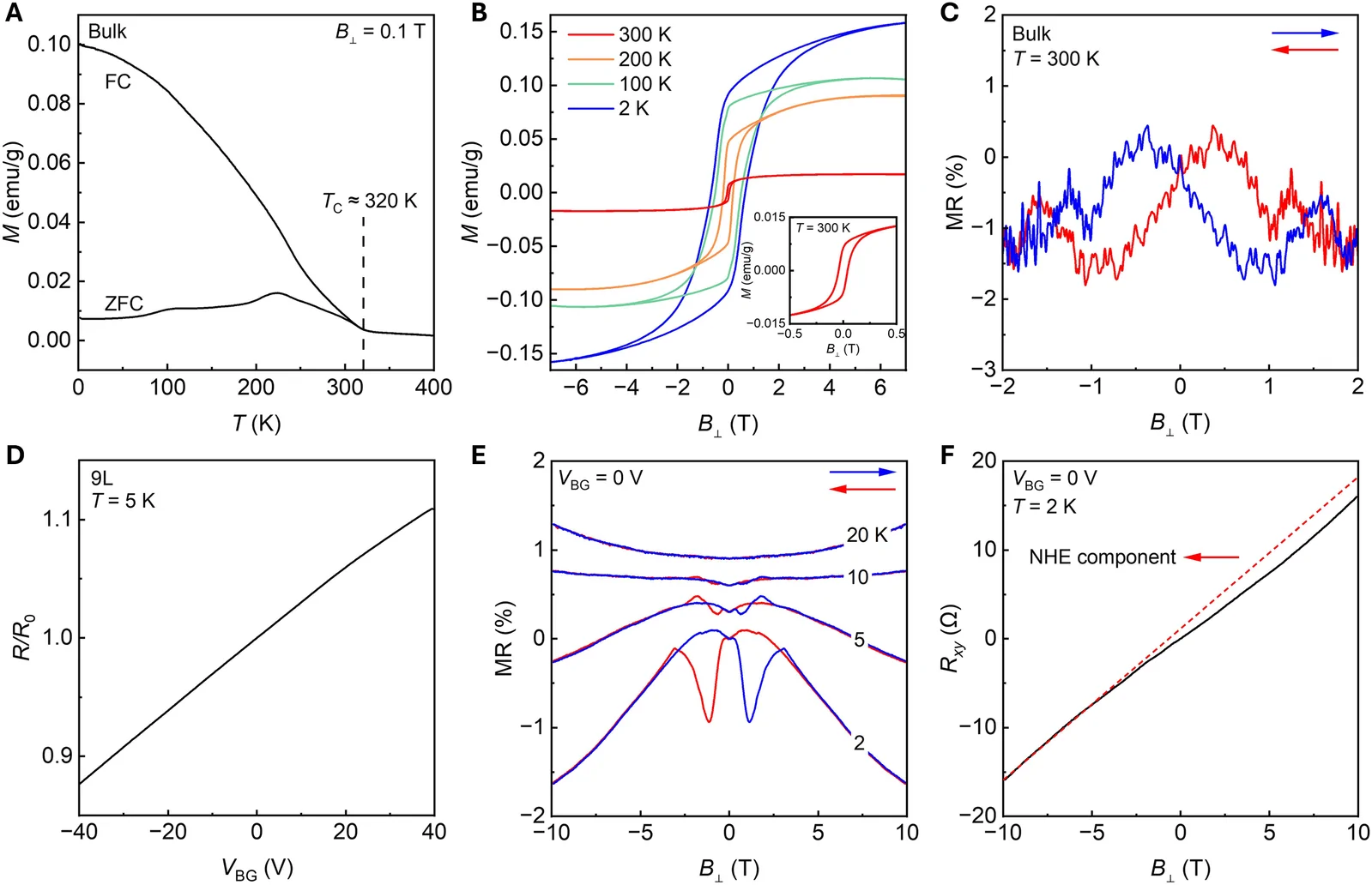
Magnetic and transport characteristics of thick Fe-doped PtSe_2_ devices. (**A**) *M*-*T* curves of bulk Fe-doped PtSe_2_ sample measured during field-cooled (FC) and zero-field-cooled (ZFC). The dashed line illustrates where the FM ordering transition occurs. (**B**) *M*-*H* curves of bulk Fe-doped PtSe_2_ sample at 2, 100, 200, and 300 K. Inset: Zoomed-in graph of *M*-*H* curves at 300 K. emu, electromagnetic unit. (**C**) MR ratio as a function of magnetic field of the bulk Fe-doped PtSe_2_ device at RT. Blue (red) curve represents magnetic field sweep direction from −2 to 2 T (2 to −2 T). (**D**) *R*-to-*R*_0_ ratio as a function of back-gate voltage of 9L Fe-doped PtSe_2_ device. *R*_0_ represents channel resistance at zero back-gate voltage, and the plot shows the limited gate tunability of the 9L device. (**E**) MR ratio as a function of magnetic field of the 9L Fe-doped PtSe_2_ device at different low temperatures. Blue (red) curve represents magnetic field sweep direction from −10 to 10 T (10 to −10 T). (**F**) *R_xy_* as a function of magnetic field of the 9L Fe-doped PtSe_2_ device at 2 K. The red dashed line represents the normal Hall effect (NHE) component, showcasing the nonlinearity of the measured Hall resistance.

Bulk Fe-doped PtSe_2_ crystals exhibit clear signatures of ferromagnetism, as revealed by superconducting quantum interference device (SQUID) magnetometry on optimized samples. Temperature-dependent magnetization under a 0.1 T applied field shows a pronounced FM transition with a Curie temperature of ∼320 K in zero-field-cooled and field-cooled measurements (Fig. 2A). Complementary *M*-*H* loops measured along the *c* crystallographic axis confirm robust FM hysteresis even at RT (Fig. 2B). Based on the Fe concentration of ∼5.5% determined from bulk EDS analysis, the magnetic moment is estimated to be ∼0.17 μ_B_ per Fe atom at 2 K (see Materials and Methods). This value is substantially smaller than that of metallic Fe (2.2 μ_B_/Fe) and also lower than the local moment predicted by our DFT calculations (1.72 μ_B_/Fe), which is inconsistent with ferromagnetism arising from Fe clustering. Such reduced effective moments per dopant atom are commonly observed in dilute magnetic systems such as Mn-doped ZnO (*42*), Co-doped TiO_2_ (*43*) and Fe-doped SnS_2_ (*44*). The FM nature of the bulk crystal is further supported by room-temperature magnetoresistance measurements, which show clear hysteresis centered at zero magnetic field (Fig. 2C).

Next, we examine whether the FM order observed in bulk persists upon reducing the thickness toward the 2D limit (*2*). To this end, we performed magnetotransport measurements on a relatively thick 9L Fe-doped PtSe_2_ device, which remains metallic and shows weak gate dependence (Fig. 2D). As shown in Fig. 2E, the magnetoresistance exhibits a clear hysteresis centered at zero magnetic field, indicating the presence of FM coupling; however, this hysteresis is only observed up to ∼20 K, revealing a strong suppression of the FM order compared to the bulk. This behavior is consistent with weak magnetic anisotropy observed in our devices (fig. S8) (*2*, *45*). The corresponding Hall resistance shows a predominantly linear field dependence with only a weak nonlinear contribution at low fields (Fig. 2F), consistent with a reduced net magnetization in this thickness regime (*46*, *47*). Similar low-temperature FM magnetotransport signatures are observed in 7L and 8L devices (figs. S6 and S7), indicating that FM coupling persists throughout the metallic few-layer regime down to seven layers.

### Gate-tunable antiferromagnetism in 2D semiconducting regime

**Fig. 3.**
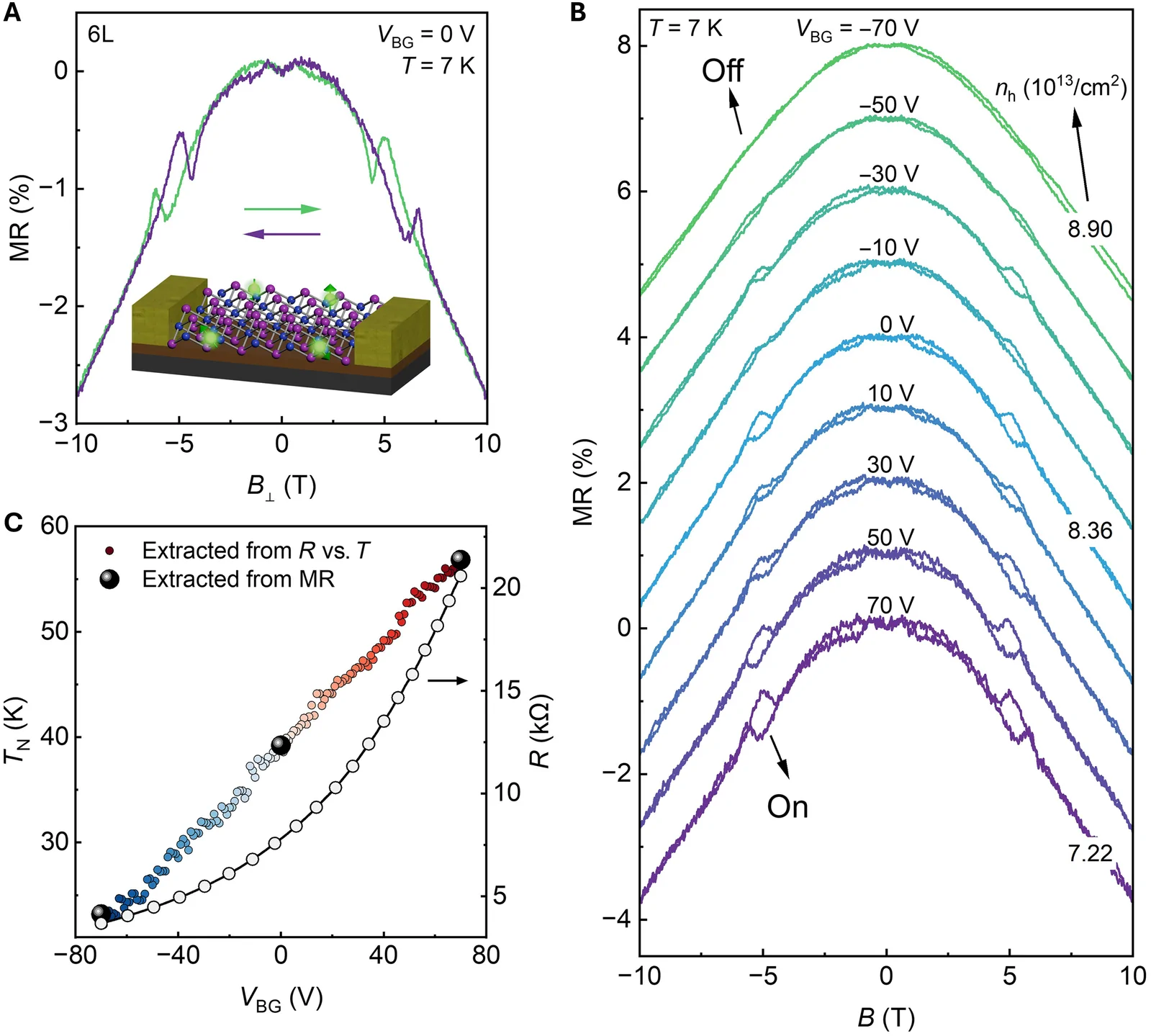
Transport characteristics of 6L Fe-doped PtSe_2_ device. (**A**) The representative MR ratio as a function of magnetic field at 7 K and zero back-gate voltage. Inset: Schematic of the Fe-doped PtSe_2_ device layout with gold contacts and SiO_2_/Si substrates. The green, blue, and purple balls represent Fe, Pt, and Se atoms, respectively. (**B**) MR ratio as a function of magnetic field at different back-gate voltages ranging from −70 to 70 V at 7 K. *n*_h_ represents hole concentration values. (**C**) Left *y* axis: Extracted Néel temperatures from temperature-dependent resistance measurements (small scatter) and MR measurements (big scatter) as a function of back-gate voltage. Right *y* axis: Resistance as a function of back-gate voltage at 7 K.

We next turn to thinner exfoliated devices to examine how the magnetic order evolves as the system approaches thinner limit, beginning with a 6L Fe-doped PtSe_2_ device that lies near the semiconducting-to-metallic crossover of PtSe_2_ (*30*). The 6L device remains sufficiently conductive for reliable magnetotransport measurements while exhibiting appreciable gate tunability, making it particularly suitable for investigating carrier-dependent magnetic behavior. Figure 3A presents the MR ratio as a function of out-of-plane magnetic field at *T* = 7 K under zero back-gate voltage (*V*_BG_ = 0). A pronounced hysteresis emerges near ±5 T, indicative of field-driven magnetic reorientation, a characteristic feature of metamagnetic transitions in antiferromagnets. The MR response is negative with increasing field, symmetric with respect to field polarity, and persists at high fields, all hallmarks of AFM order. Similar magnetotransport signatures, including sharp finite-field hysteresis associated with metamagnetic reorientation, have been widely reported in well-established antiferromagnets such as NiSi (*48*), CeSb (*49*), EuIn_2_As_2_ (*50*), and EuAg_4_As_2_ (*51*), whose AFM order has been independently confirmed by neutron scattering. The Hall resistance *R_xy_* remains linear over the full magnetic-field range (fig. S9), indicating the absence of a sizable anomalous Hall contribution. This observation is consistent with an AFM-dominated state lacking significant momentum-space Berry curvature, as expected for collinear or weakly canted antiferromagnets without nontrivial spin textures (*52**–**55*). We note that similar negative MR can also occur in disordered magnetic states (e.g., spin glass or superparamagnetism); however, the sharp finite-field hysteresis centered near ±5 T observed here, together with the nonsaturating high-field MR and the absence of a resolvable hysteretic Hall signal, is most consistent with AFM metamagnetic reorientation. Last, we note that, in addition to the dominant finite-field hysteresis, a much weaker hysteresis centered at *B* = 0 T is also observed in the 6L device. Such a weak near-zero-field hysteresis may arise from uncompensated AFM moments due to sublattice imbalance (*48*) or from a field-induced canted AFM state (*9*). Given that the 6L device lies near the transition from semiconducting to semimetallic behavior and the similarity of the characteristic fields to those observed in thicker FM samples, this weak feature may also reflect a residual FM component inherited from the metallic regime.

To examine electrostatic control of magnetic order, we perform MR measurements across a broad range of back-gate voltages (*V*_BG_ = −70 to +70 V). As shown in Fig. 3B, at 7 K, both the hysteresis and overall MR amplitude are negligible at −70 V, where the system is strongly hole-doped and metallic. As *V*_BG_ increases, reducing the hole concentration, the Hall carrier density decreases from 8.9 × 10^13^ cm^−2^ at *V*_BG_ = −70 V to 7.2 × 10^13^ cm^−2^ at *V*_BG_ = +70 V (fig. S9), the magnetic response becomes progressively more pronounced, reaching a maximum MR change of 0.67% at +70 V. This gate-induced activation of hysteresis demonstrates that AFM ordering can be switched on through electrostatic modulation. We define the Néel temperature of our Fe-doped device as the temperature at which magnetic hysteresis in the MR disappears, despite the persistence of negative MR (figs. S10 and S11). Based on this criterion, *T*_N_ increases from 25 K at *V*_BG_ = −70 V to 55 K at *V*_BG_ = +70 V (fig. S12). These values are independently corroborated by inflection-point analysis of temperature-dependent resistance curves (fig. S13) (*56*), establishing a clear inverse relationship between *T*_N_ and carrier concentration, as summarized in Fig. 3C.

**Fig. 4.**
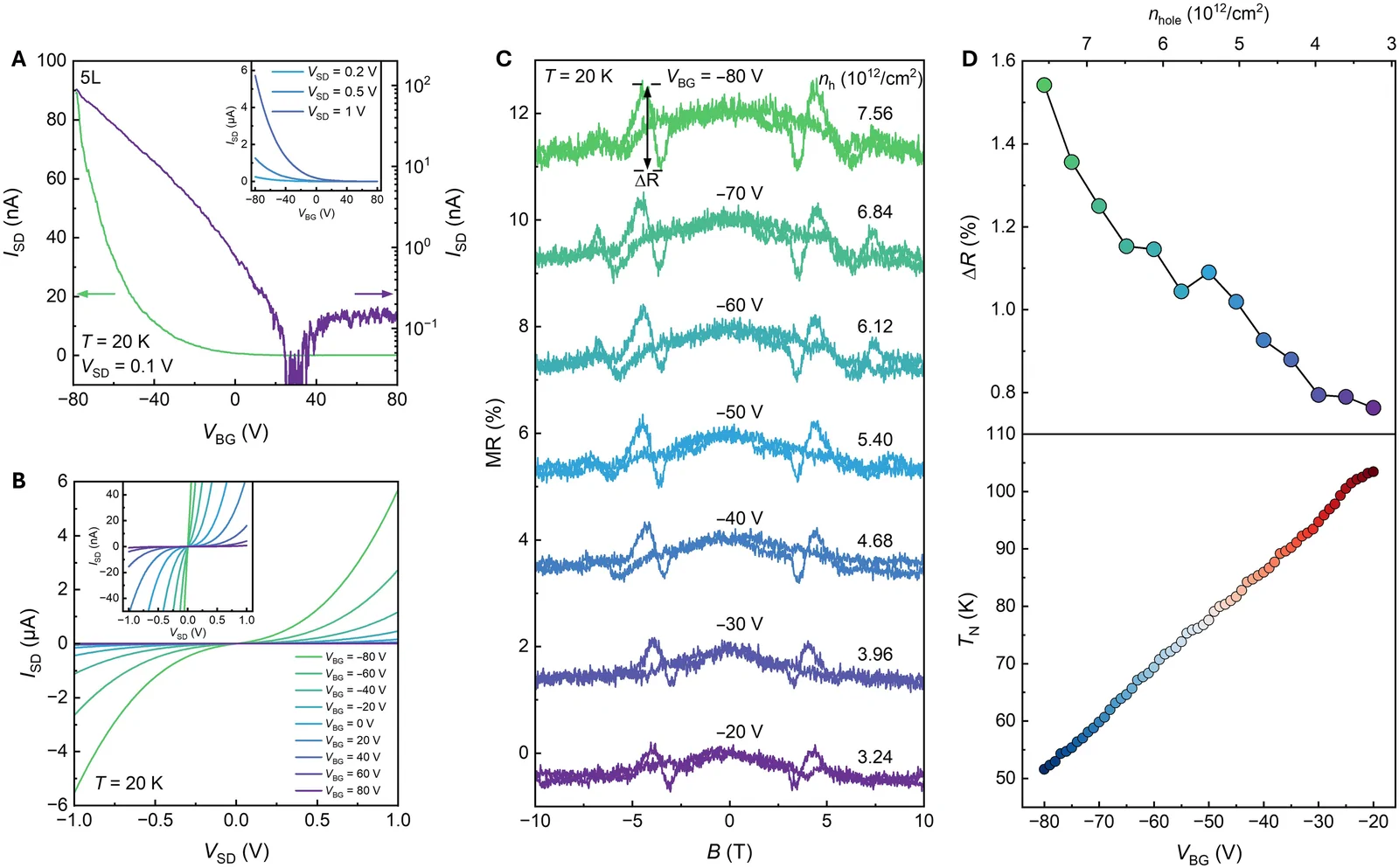
Transport characteristics of 5L Fe-doped PtSe_2_ device. (**A**) Source-drain current as a function of back-gate voltage at 20 K, measured at a bias of 0.1 V (linear scale in left *y* axis and log_10_ scale in right *y* axis). Inset: Source-drain current as a function of back-gate voltage at 20 K, measured at a bias of 0.2, 0.5, and 1 V. (**B**) Source-drain current as a function of source-drain bias voltage at 20 K, measured at different back-gate voltages ranging from −80 to 80 V. Inset: Zoomed-in graph of source-drain current at different back-gate voltages. (**C**) MR ratio as a function of the magnetic field at different back-gate voltage ranging from −80 to −20 V at 20 K. Δ*R* represents the MR difference of hysteretic part between the two sweep directions. *n*_h_ represents hole concentration values. (**D**) Δ*R* (top) and extracted Néel temperatures from temperature-dependent resistance measurements (bottom) as a function of back-gate voltage and hole concentration.

To further explore the semiconducting regime, we investigate a 5L Fe-doped PtSe_2_ device. Similar to pristine 5L PtSe_2_ (fig. S14), the Fe-doped device exhibits clear semiconducting transport behavior, with an on/off current ratio exceeding 10^3^ over a *V*_BG_ range of −80 to +80 V (Fig. 4, A and B). MR measurements at 20 K (Fig. 4C) again reveal pronounced hysteresis near ±5 T, consistent with field-induced reorientation of antiferromagnetically coupled moments. The hysteresis cannot be directly probed when the device is in its off-state (*V*_BG_ > 25 V) due to large background signal but becomes increasingly prominent as the system is hole-doped (*V*_BG_ < −20 V). To quantify the strength of this hysteresis, we define Δ*R* as the difference in MR between the two sweep directions within the hysteretic region. Although the measured Δ*R* decreases as the carrier concentration is reduced (Fig. 4D, top), this trend is likely influenced by the rapidly increasing contact resistance and Schottky barrier effects as the device approaches its off state. These transport-related effects obscure the intrinsic interaction between carriers and magnetic impurities and, therefore, limit the use of Δ*R* as a quantitative measure of AFM strength in the low-carrier-density regime. By contrast, the Néel temperature extracted from temperature-dependent transport measurements (figs. S15 and S16) (*56*) increases systematically from ∼52 to 105 K as the carrier concentration decreases, further supporting the strong inverse correlation between *T*_N_ and *n* (Fig. 4D, bottom). Together, the 5L and 6L results demonstrate that AFM order persists in the semiconducting regime and can be continuously modulated, or even switched on and off, by gate voltage.

### Carrier-mediated magnetic ordering transitions

**Fig. 5.**
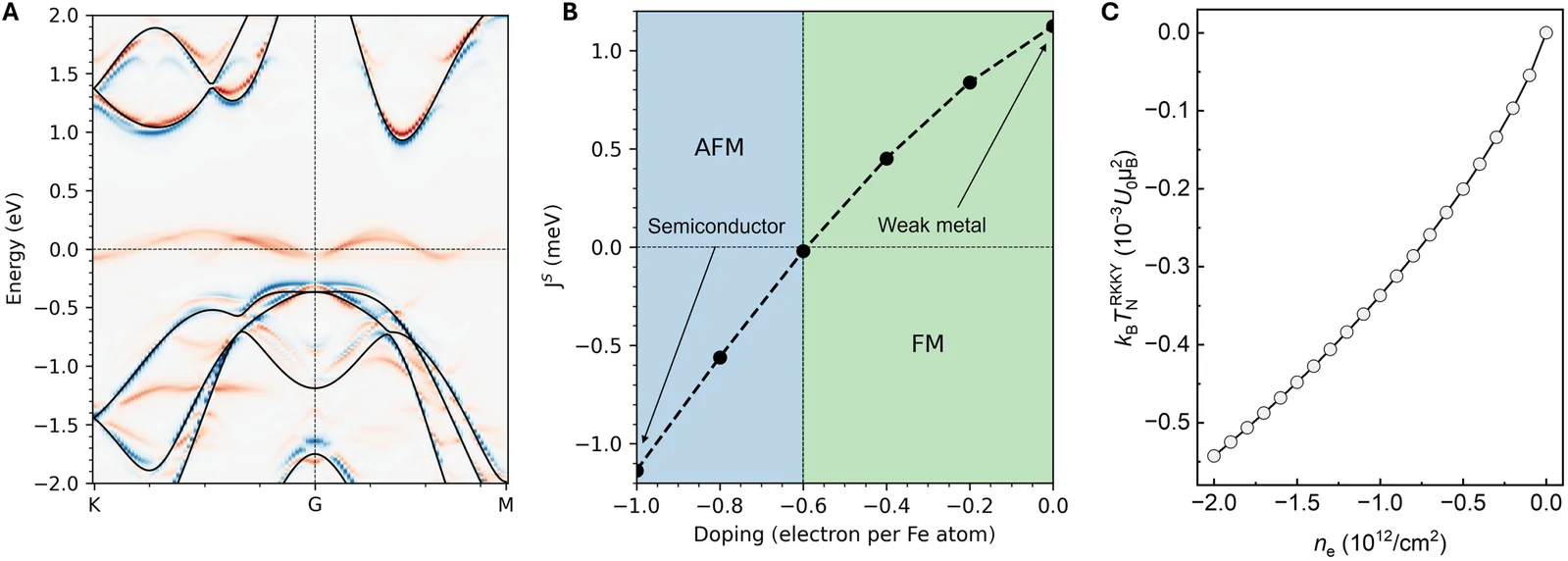
Computational analysis. (**A**) Spin-resolved unfolded band structure of the Fe-doped 3 × 3 supercell PtSe_2_ monolayer, where black solid lines denote the band structure of pristine PtSe_2_ monolayer. (**B**) Magnetic phase diagram illustrating the doping dependence of superexchange Fe-Fe coupling (*J* ^S^) in the Fe-doped 3 × 3 supercell PtSe_2_ monolayer. A transition from FM to AFM ordering is predicted as the system shifts from a weak metal to a semiconductor regime. (**C**) Nearly linear dependence of Néel temperature renormalization on hole doping, mediated by 2D RKKY interaction. Results are derived from mean-field calculations.

To provide insights into the microscopic origin of magnetism and observed gate tunability in thin Fe-doped PtSe_2_ devices, we begin by modelling our system as a 3 × 3 supercell of monolayer PtSe_2_ with a single Fe atom substituting a Pt site. DFT calculations of the spin-resolved unfolded band structure (Fig. 5A) reveal that the spin-polarized Fe bands exist near the Fermi level, which is partially responsible for the MR in the magnetotransport measurements. The role of the on-site Coulomb interaction on the Fe d-states is investigated by performing DFT + *U* calculations for *U* values up to 4 eV (fig. S17A). In this range, the impact on the low-energy band structure and on the magnetic moments (see Materials and Methods) is negligible: The overall band topology and spin polarization near the Fermi level remain essentially unchanged. Because the effective Fe concentration in a 3 × 3 supercell (11.1%) is higher than the experimentally determined value (∼5.5%), we further performed calculations using larger 4 × 4 (6.25%) and 5 × 5 (4%) supercells (fig. S17, B and C). The qualitative features, including the Fe local moment and spin polarization, remain robust across these supercell sizes. We note that we limit our calculations to monolayer as standard DFT underestimates the bandgap of multilayer PtSe_2_ (*57*), leading to unreliable impurity-level positioning, whereas monolayer provides a well-defined electronic structure suitable for capturing the essential magnetic interactions (see fig. S18 for bilayer and bulk cases).

For the evaluation of the magnetic exchange mechanism inside, we use the Wannier90 package (*58*) to construct a tight-binding Hamiltonian and TB2J package (*59*) to extract Fe-Fe exchange parameters via the magnetic force theorem (*59*, *60*). Without considering the gate-induced doping into the PtSe_2_ valence bands, the carrier-mediated RKKY interaction between Fe local moments is strongly suppressed because it is proportional to the spin susceptibility of the itinerant electrons, which vanishes in a gapped system. In contrast, superexchange via virtual hopping processes through Se/Pt sites does not require carriers at the Fermi level and, therefore, remains the only effective coupling mechanism between dilute Fe moments. From our DFT-based mapping of the magnetic interactions, the nearest-neighbor exchange is found to be the only appreciable coupling, whereas the further-neighbor terms (at least up to fourth to fifth neighbors) are numerically negligible and do not modify the magnetic phase diagram within our accuracy (fig. S19). As shown in Fig. 5B, the nature of Fe-Fe superexchange interaction evolves with carrier concentration: FM superexchange dominates at low hole doping (<60% of the Fe concentration), whereas AFM superexchange starts to appear at semiconducting regime (60 to 100%) in which such AFM coupling is stable even under the consideration of Hubbard *U* correction (fig. S20). RKKY-type interactions emerge upon introducing excessive holes (>100%) into the PtSe_2_ valence bands, reflecting a transition from localized to itinerant exchange mechanisms. These calculation results underscore the critical role of carrier concentration in determining the magnetic ground state, especially in thin Fe-doped PtSe_2_. Accordingly, complete closure of the bandgap above six layers in PtSe_2_ enhances carrier screening, transforming a system where superexchange and RKKY interactions compete into one mediated entirely by RKKY, which is possibly responsible for the origin of the FM order in thick samples.

To investigate the gate-tunable Néel temperature observed from our transport measurements in 5L and 6L Fe-doped PtSe_2_ devices, we use a mean-field approach in which the total Néel temperature is treated as the sum of contributions from superexchange ($T_{N}^{S}$) and RKKY exchange ($T_{N}^{\text{RKKY}}$) (see Materials and Methods). Numerical calculations predict an almost linear relationship between the doping level and $T_{N}^{\text{RKKY}}$ (Fig. 5C), which is consistent with our experimental data: *T*_N_ increases with increasing hole concentration in 5L and 6L devices.

## DISCUSSION

Our study establishes Fe-doped PtSe_2_ as a true 2D DMS, with STEM analysis confirming successful Fe substitution into Pt sites with uniform distribution and no evidence of clustering. The unique electronic structure of PtSe_2_ (*31*), combined with efficient electrostatic control at the 2D limit, enables modulation of both the strength and nature of magnetic ordering. The electrostatically tunable AFM order in 5L and 6L devices with the inverse relationship between *T*_N_ and carrier concentration and the emergence of FM behavior in the thicker regime together highlight Fe-doped PtSe_2_ as a promising platform for exploring carrier-mediated magnetism in two dimensions through both gate voltage and layer thickness. Further enhancement of *T*_N_ may be achieved by engineering the Fe concentration and by exploring thinner devices with larger band gaps (*31*), potentially extending the AFM order toward RT, particularly in the monolayer limit. In addition, gate-induced inversion symmetry breaking in few-layer samples could amplify the intrinsic Rashba spin-orbit coupling of PtSe_2_, enabling spin-orbit torque functionality within a single material, without the need for an external heavy-metal layer (*61*, *62*). These features position Fe-doped PtSe_2_ as a promising platform for next-generation, energy-efficient spintronic devices based on electrically reconfigurable AFM order.

## MATERIALS AND METHODS

### Crystal growth and sample preparation

Fe-doped PtSe_2_ were made directly from elements in quartz ampoule by crystallization of molten flux. For the synthesis, platinum (99.99%, sponge, SurePure, USA), iron (99.9%, −100 mesh, Alfa Aeasar, Germany), and selenium (99.9999%, granules of 2 to 4 mm, Wuhan Xinrong New Materials Co. China) were placed in quartz ampoule (25 mm by 100 mm, 3-mm wall thickness) and melt sealed under high vacuum (< 1 × 10^−3^ Pa using oil diffusion pump and liquid nitrogen trap) using oxygen hydrogen torch. For the synthesis, 3 g of batch of PtSe_2_ with 2 at % excess of selenium was used. The ampoule was placed in muffle furnace and heated to 800°C in 4 hours, subsequently to 1000°C in 12 hours, and lastly to 1270°C in 2 hours. Subsequently, the ampoule was cooled to 1200°C in 2 hours and then by 5°C/min to RT. The ampoule was open in argon-filled glove box, and formed PtSe_2_-based crystals were mechanically separated.

Thin flakes of Fe-doped PtSe_2_ were prepared via the standard Scotch-tape mechanical exfoliation method using Nitto-Denko adhesive tape. Before exfoliation, SiO_2_/Si substrates were treated with O_2_ plasma to activate the surface, followed by brief heating on a hot plate at 100°C for 30 min after crystal placement to improve adhesion and enhance the yield of ultrathin flakes. Electrical contacts that contain 100-nm Au with a 2-nm Ti adhesion layer were defined by the electron beam lithography followed by the electron beam evaporation. The metallization process was completed using a liftoff process in an acetone/isopropanol mixture, followed by nitrogen drying.

### STEM characterization

The bilayer Fe-doped PtSe_2_ was prepared by RT transfer of exfoliated crystals on polydimethylsiloxane onto a holey SiN transmission electron microscopy substrate with a hole size of ∼1 μm. The 20-nm–thick Fe-doped PtSe_2_ sample were coated with a platinum protection film, after which cross-sectional samples were cut with a Gallium ion beam in a ThermoFisher Helios 5 DualBeam system, finishing the ion milling at a gentle 1 keV to minimize surface amorphization.

Aberration-corrected STEM imaging was done with a JEOL ARM200, operated at 200 keV with an ASCOR aberration-corrector and a cold field emission source. A convergence semiangle of 31 mrad was used, with annular dark-field inner collection angles of ∼60 mrad and an on-axis bright field detector. STEM-EDS mapping was performed using a Thermo Fisher Spectra STEM, operated at 200 keV beam energy. A convergence semiangle of 27 mrad was used, together with a DualX EDS detector.

### Measurement

Cryogenic measurements were conducted using a closed-cycle Oxford Instruments cryomagnetic system with a base temperature of ∼1.5 K. A Keithley 2450 SourceMeter was used to apply bias through the SiO_2_ gate dielectric. Charge transport measurements were carried out using a Stanford Research SR830 lock-in amplifier at low frequency with excitation currents ranging from 0.01 to 1 μA. Another Keithley 2450 SourceMeter was used to apply source-drain bias and read the resulting source-drain current in resistive devices.

### Calculations

The calculations were performed using the GPAW code (*63*) with the double-zeta polarized linear combination of atomic orbitals basis set (*64*) and the local spin-density approximation for the exchange-correlation functional. A vacuum spacing of 8 Å was included to minimize interlayer interactions. The relaxed structure of a 3 × 3 supercell of monolayer PtSe_2_ with a single Fe atom substituting a Pt site is adopted for main discussion.

DFT calculations yield a magnetic moment per Fe atom (μ_B_/Fe) obtained by integrating the spin density within Fe-centered atomic regions. In the absence of Hubbard *U* correction, we obtain 1.72 μ_B_/Fe for the 3 × 3 supercell (fig. S21). For a Hubbard interaction *U* = 4 eV applied to the Fe d-states, we obtain local moments of 2.72, 2.72, and 2.71 μ_B_/Fe in the 3 × 3, 4 × 4, and 5 × 5 supercell with a single substitutional Fe atom, respectively. The variation with supercell size remains within 0.01 _μ_B, demonstrating the magnetism is predominantly localized on the Fe sites, with only small induced moments on neighboring Se atoms. The dependence of μ_B_/Fe on both carrier doping and Hubbard *U* is shown in fig. S21.

The large spin ($S>2\mu_{B}$) of Fe moments allows for a classical treatment of the spins, which we model as Heisenberg spins governed by the Hamiltonian

$$H=\sum_{\mathrm{ij}}J\left(r_{\mathrm{ij}}\right)S_{i}\cdot S_{j}+\sum_{\left\langle \mathrm{ij}\right\rangle }J^{S}S_{i}\cdot S_{j}$$ (1)

where $r_{\mathrm{ij}}$ denotes the spatial separation between moments i and j and the interaction term

$$J\left(r_{\mathrm{ij}}\right)=J_{\text{ex}}^{2}\chi \left(r_{\mathrm{ij}}\right)$$ (2)

describes the RKKY interaction, with $J_{\text{ex}}$ being the exchange coupling constant between a localized magnetic impurity $S_{i}$ and the itinerant electron spin $s\left(r\right)=\frac{\hbar }{2}\delta \left(r\right)\sigma$, given by

$$H_{\text{ex}}=-J_{\text{ex}}\sum_{i}S_{i}\cdot s\left(r_{i}\right)$$ (3)

The polarizability of 2D Fermi gas is expressed as (*65*)

$$\chi \left(r\right)=-D\left(E_{F}\right)k_{F}^{2}\left[J_{0}\left(k_{F}r\right)Y_{0}\left(k_{F}r\right)+J_{1}\left(k_{F}r\right)Y_{1}\left(k_{F}r\right)\right]$$ (4)

where $J_{0,1}$ and $Y_{0,1}$ are the Bessel functions of the first and second kind, respectively; $D\left(E_{F}\right)=\frac{|m|}{\pi \hbar^{2}}$ represents the electron density of states; and $k_{F}=\sqrt{|n_{e}|/\pi }$ denotes the Fermi wavevector, with $n_{e}$ being the charge carrier concentration. By substituting these expressions into Eq. 2, we obtain the following form for the RKKY interaction

$$J\left(r_{\mathrm{ij}}\right)=-U_{0}k_{F}^{2}\left[J_{0}\left(k_{F}r\right)Y_{0}\left(k_{F}r\right)+J_{1}\left(k_{F}r\right)Y_{1}\left(k_{F}r\right)\right]$$ (5)

where $U_{0}=J_{\text{ex}}^{2}\frac{|m|}{\pi \hbar^{2}}$

In addition to the RKKY interaction, the Hamiltonian includes a superexchange term, where $J^{S}$ represents the local AFM superexchange coupling. This interaction is relevant only for the neighboring impurities and is determined through ab initio calculations.

For a system described by the Heisenberg Hamiltonian, the mean-field Néel temperature *T*_N_ can be calculated using the virtual crystal approximation as (*66*)

$$T_{N}k_{B}=-\frac{S\left(S+1\right)}{3}\sum_{j}J\left(r_{\mathrm{ij}}\right)$$ (6)

where S is the spin quantum number and $J\left(r_{\mathrm{ij}}\right)$ represents the exchange interaction between spins at sites i and j.

Néel temperature mean-field temperature equation can be decomposed into two contributions, yielding Eq. 7 for the critical temperature

$$T_{N}=T_{N}^{\text{RKKY}}+T_{N}^{S}$$ (7)

where $T_{N}^{\text{RKKY}}$ represents the contribution from the RKKY interaction and $T_{N}^{S}$ is the superexchange contribution.

In the limit of zero electron doping, $T_{N}^{\text{RKKY}}$ =0 due to the absence of itinerant electrons, the critical temperature reduces to $T_{N}=T_{N}^{S}$. Additionally, we assume that $T_{N}^{S}$ remains independent of electron doping as the effect of doping on the electron band structure is negligible. Numerical calculations predict an almost linear relationship between the doping level and $T_{N}^{\text{RKKY}}$, as illustrated in Fig. 5C. Therefore, we assume a linear decrease in the Néel temperature for the decreasing electron doping.

In general, magnetic anisotropy plays a crucial role in 2D magnetism (*2*). However, our test calculations of Fe-doped PtSe_2_ including spin-orbit coupling indicate that the spin-orbit–induced energy scale is very small compared to the leading exchange interactions. The corresponding magnetic anisotropy energy per Fe atom is below our numerical accuracy and does not result in a robust, well-defined easy axis. Once we incorporate this small anisotropy into the effective spin model used for the mean-field calculations, the extracted anisotropy parameters are found to be highly sensitive to numerical details and lead to unstable/unreliable mean-field results. Given that our main focus is on identifying the dominant exchange mechanism and the presence of a stable magnetic ground state, we restrict our analysis to an isotropic Heisenberg model. A fully quantitative treatment of magnetic anisotropy in Fe-doped 1T-PtSe_2_ is thus deferred to future work beyond the scope of the present study.
