# Supplementary material for: Electrical control of induced magnetism in an air-stable two-dimensional semiconductor
Source: Sci Adv. 2026 Sep 2;12(36):eaeb0659. doi: 10.1126/sciadv.aeb0659 (PMC13537247; doi:10.1126/sciadv.aeb0659)
Supplement: Supplementary file 1 [file sciadv.aeb0659_sm.pdf]

Science Advances
AAAS

Supplementary Materials for

Electrical control of induced magnetism in an air-stable two-dimensional semiconductor

Qi Zhang *et al.*

Corresponding author: Ahmet Avsar, aavsar@nus.edu.sg

Sci. Adv. **12**, eaeb0659 (2026)
DOI: 10.1126/sciadv.aeb0659

This PDF file includes:

Supplementary Text
Figs. S1 to S21

Supplementary Text

Additional characterizations

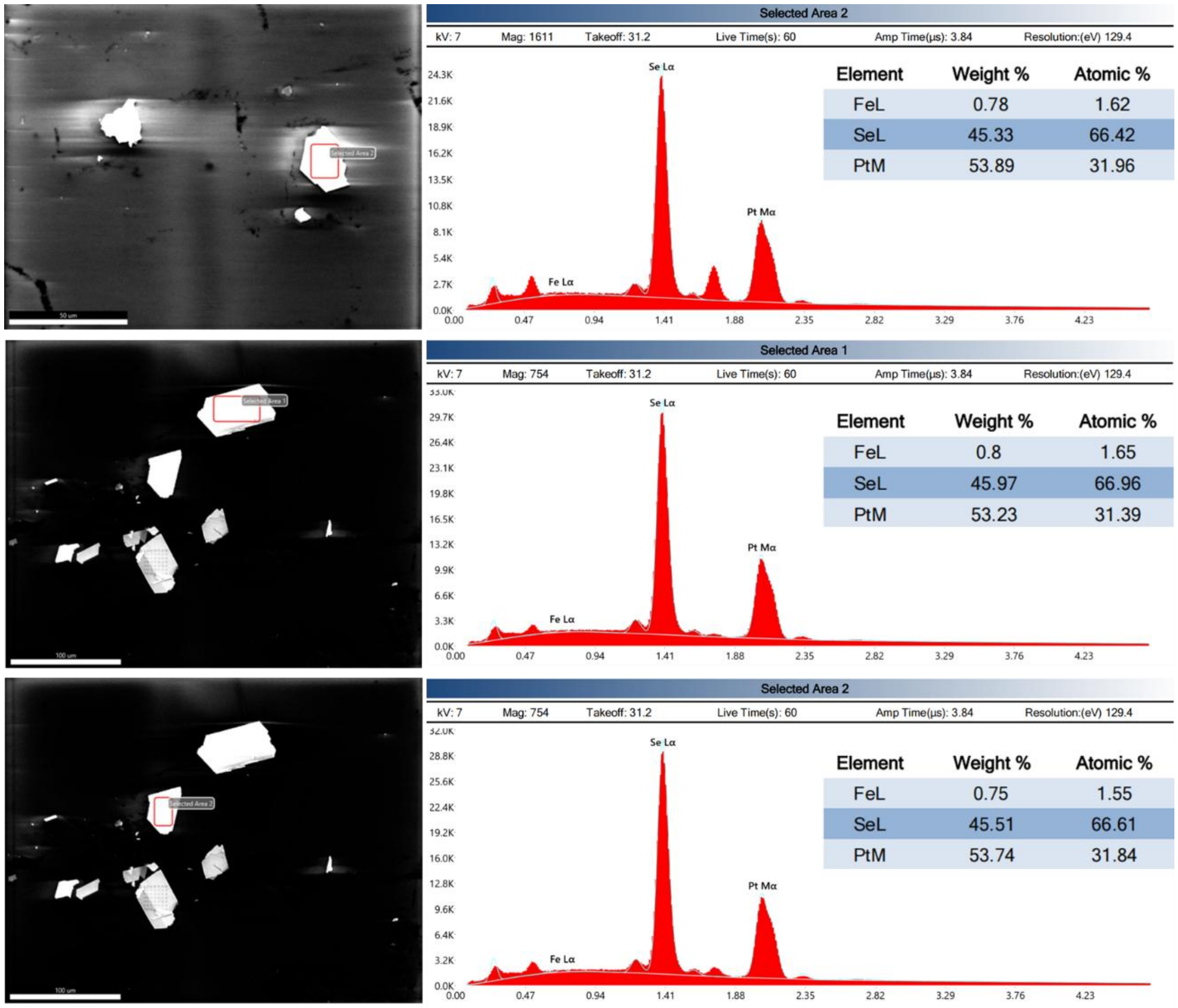

Fig. S1. The SEM-EDS spectra of three different Fe-doped $PtSe_2$ flakes.

Air-stability of Fe-doped $PtSe_2$ was investigated through surface and Raman characterization techniques. The comparative AFM images and Raman spectra of the Fe-doped $PtSe_2$ flake (Fig.1C in the manuscript) before and after 250°C annealing show no significant signs of degradation, demonstrating its air-stability.

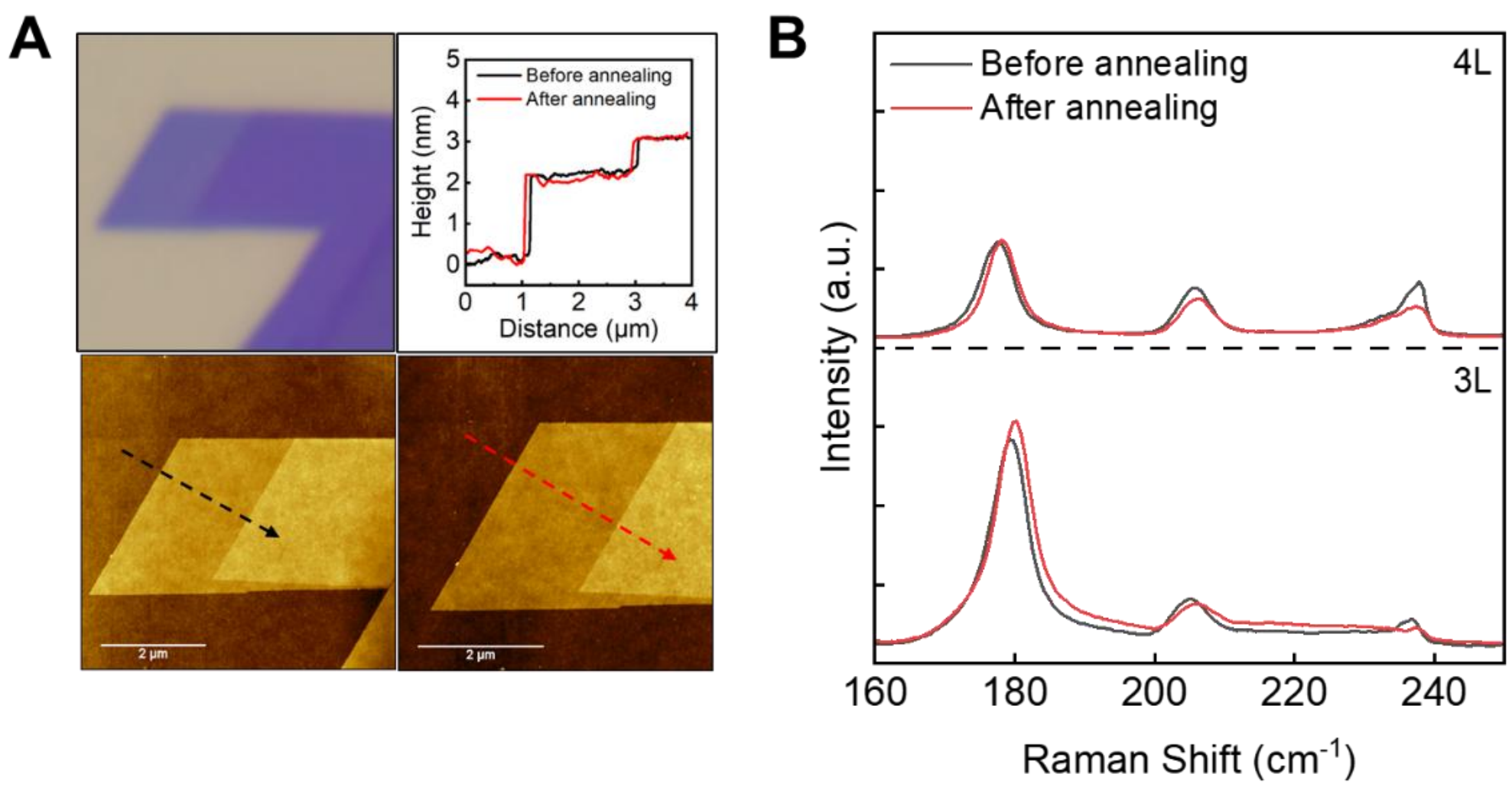

Fig. S2. Experimental demonstration of air-stability of Fe-doped $PtSe_2$. (**A**) The optical image of the Fe-doped $PtSe_2$ flake (Fig. 1C), and its AFM images taken before and after 250°C annealing with profiles of black and red arrow lines. (**B**) The Raman spectra of the Fe-doped $PtSe_2$ sample measured before and after 250°C annealing.

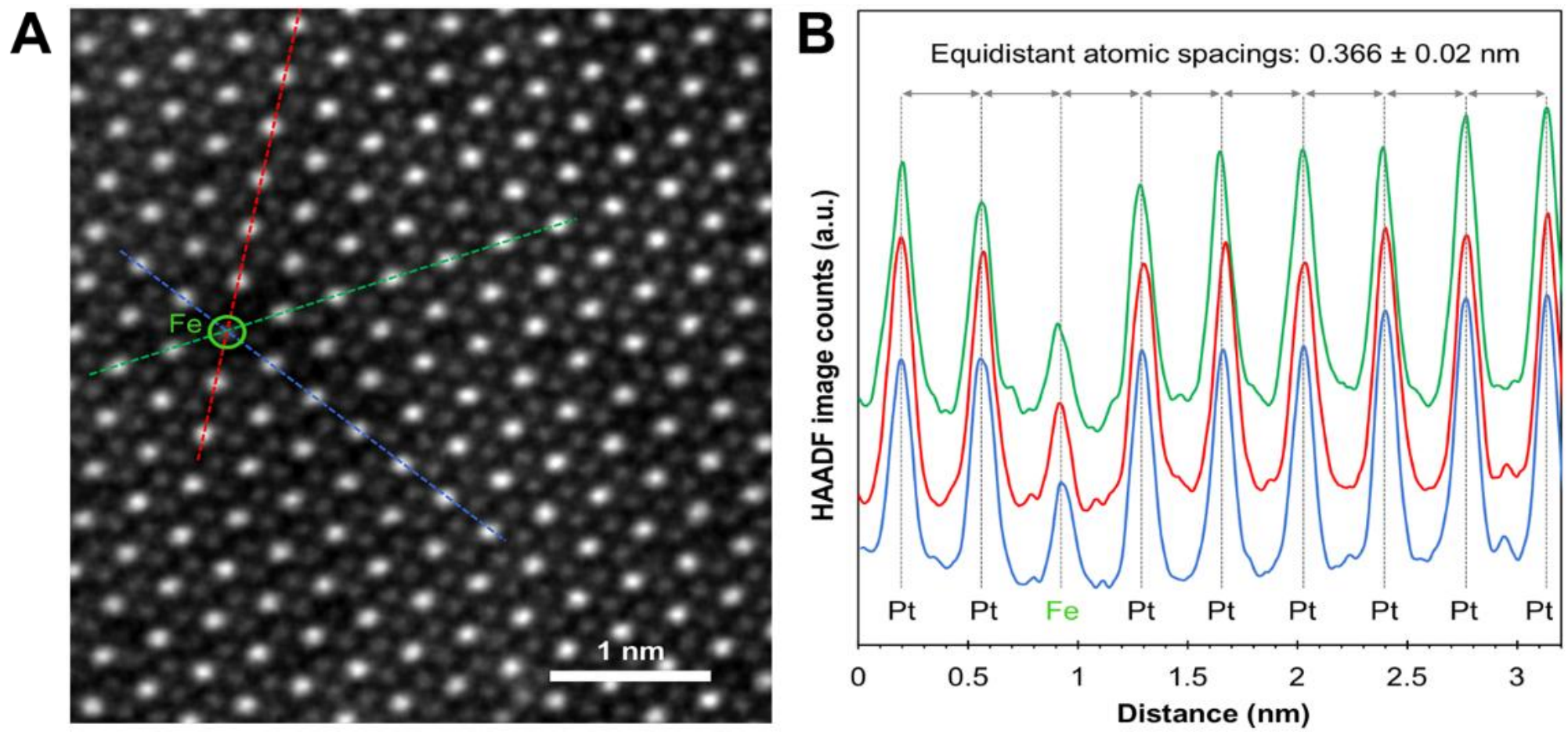

Fig. S3. Intensity profile analysis of the HAADF STEM image of the bilayer Fe-doped $PtSe_2$ flake. (**A**) Intensity profiles were taken in three directions across the Fe atom. (**B**) Intensity variations with the location of the Pt and Fe atoms indicated. The interatomic Pt-Pt and Pt-Fe distance is the same: 0.366 ± 0.02 nm, indicating that the Fe atom takes the place of the substitutionally replaced Pt atom.

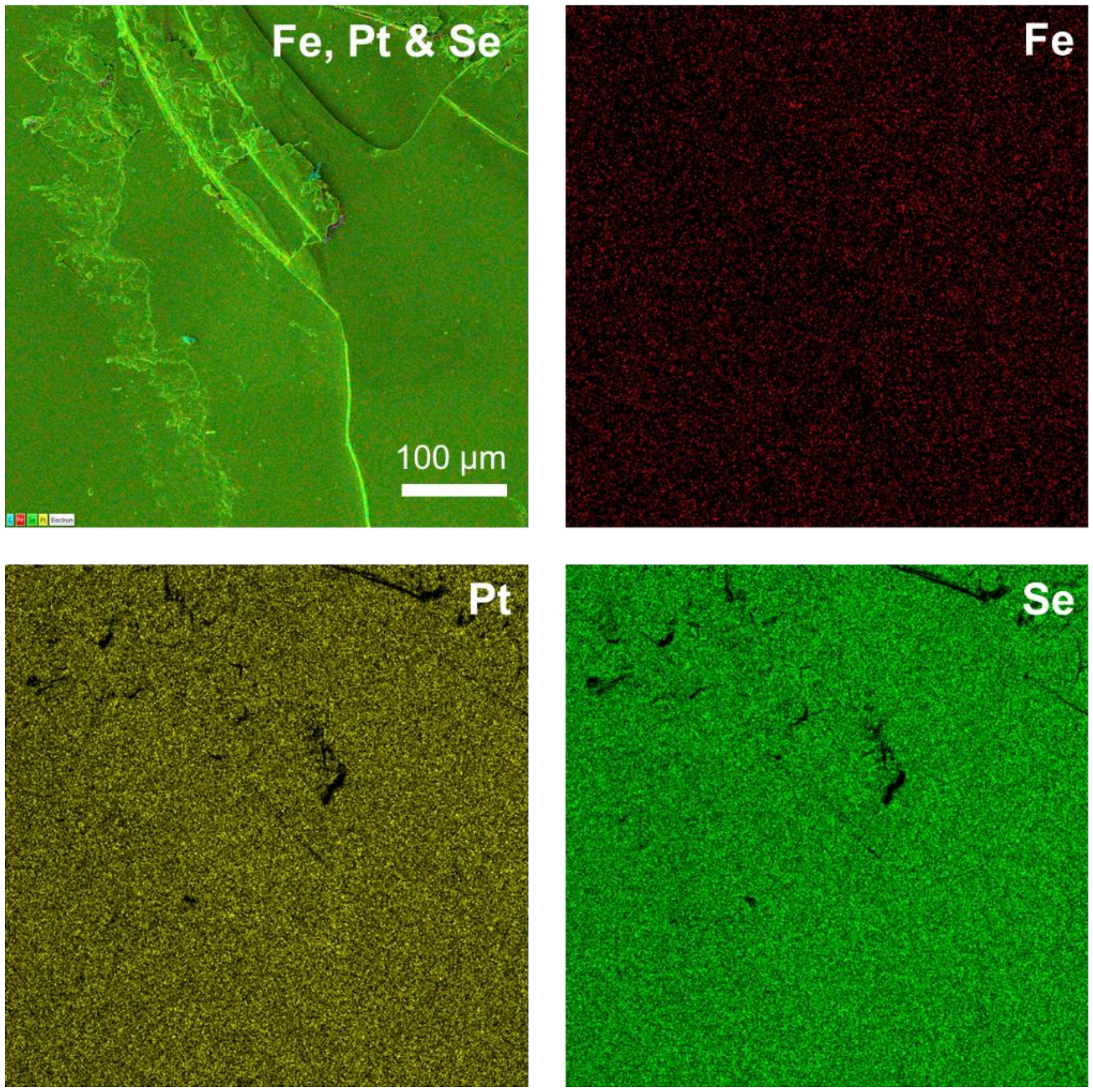

Fig. S4. SEM-EDS elemental mapping of the homogeneous distribution of Fe (red), Pt (yellow), and Se (green) across bulk Fe-doped $PtSe_2$ sample.

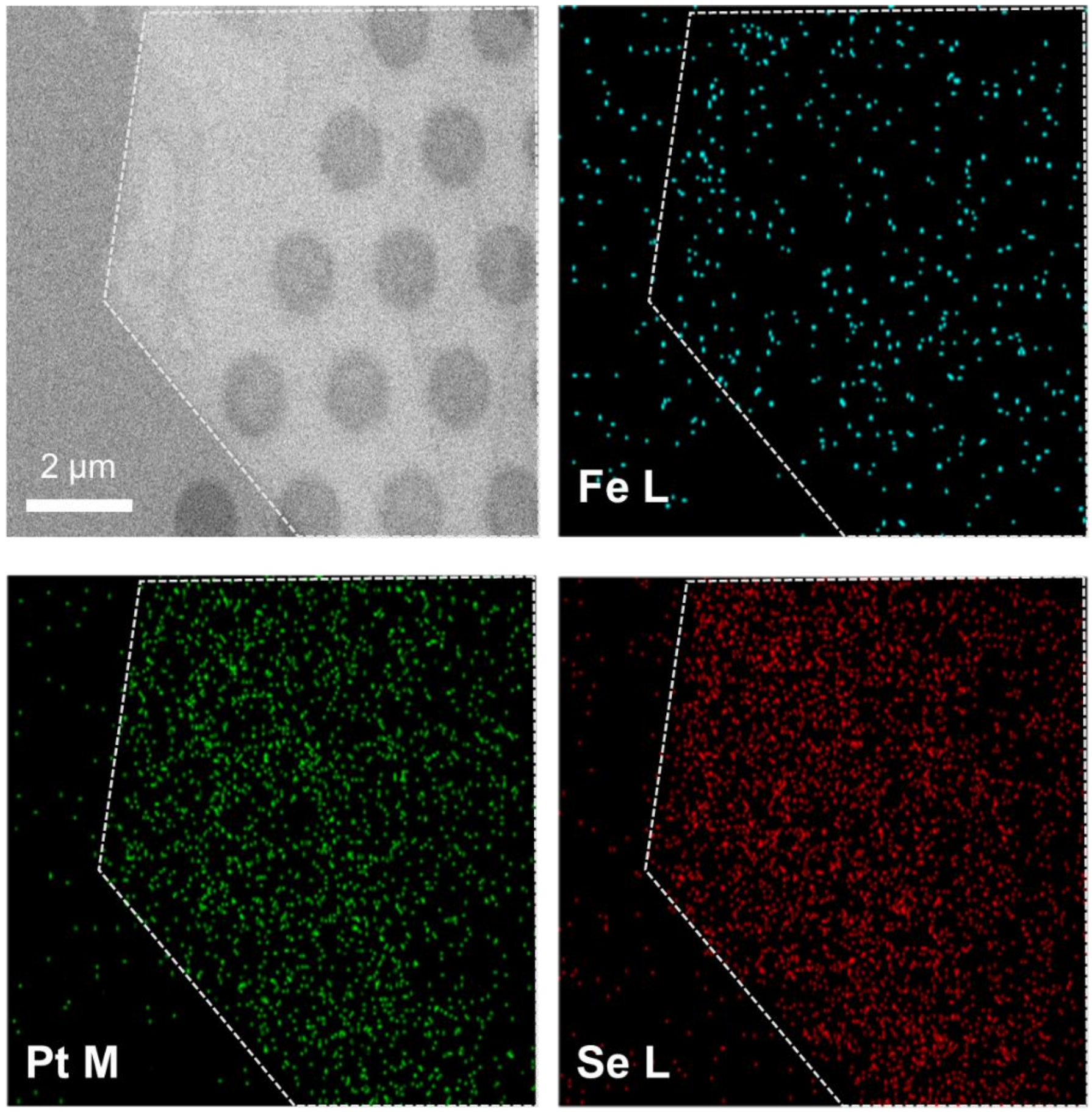

Fig. S5. STEM-EDS elemental mapping of the homogeneous distribution of Fe L (blue), Pt M (green), and Se L (red) across bilayer Fe-doped $PtSe_2$ sample.

Additional measurements on 7L, 8L, and 10L devices.

Fig. S6A shows the temperature-dependent MR ratio as a function of magnetic field of 10L device at 0 back-gate voltage, demonstrated hysteresis around zero field and $T_C \approx 20$ K.

Fig. S6B shows the R_{xy} as a function of magnetic field of 10L device at zero back-gate voltage and 2 K. The carrier concentration n_{hole} can be estimated by 2D Hall coefficient defined by $R_H = \frac{1}{n_{hole}q}$, where R_H is the slope of R_{xy} vs B, and q is elementary charge of hole. The calculated n_{hole} is about 2.36×10^{14} cm^{-2} which has 2 orders of magnitude higher carrier concentration than that of 5L semiconducting device ($\sim10^{12}$ cm^{-2}).

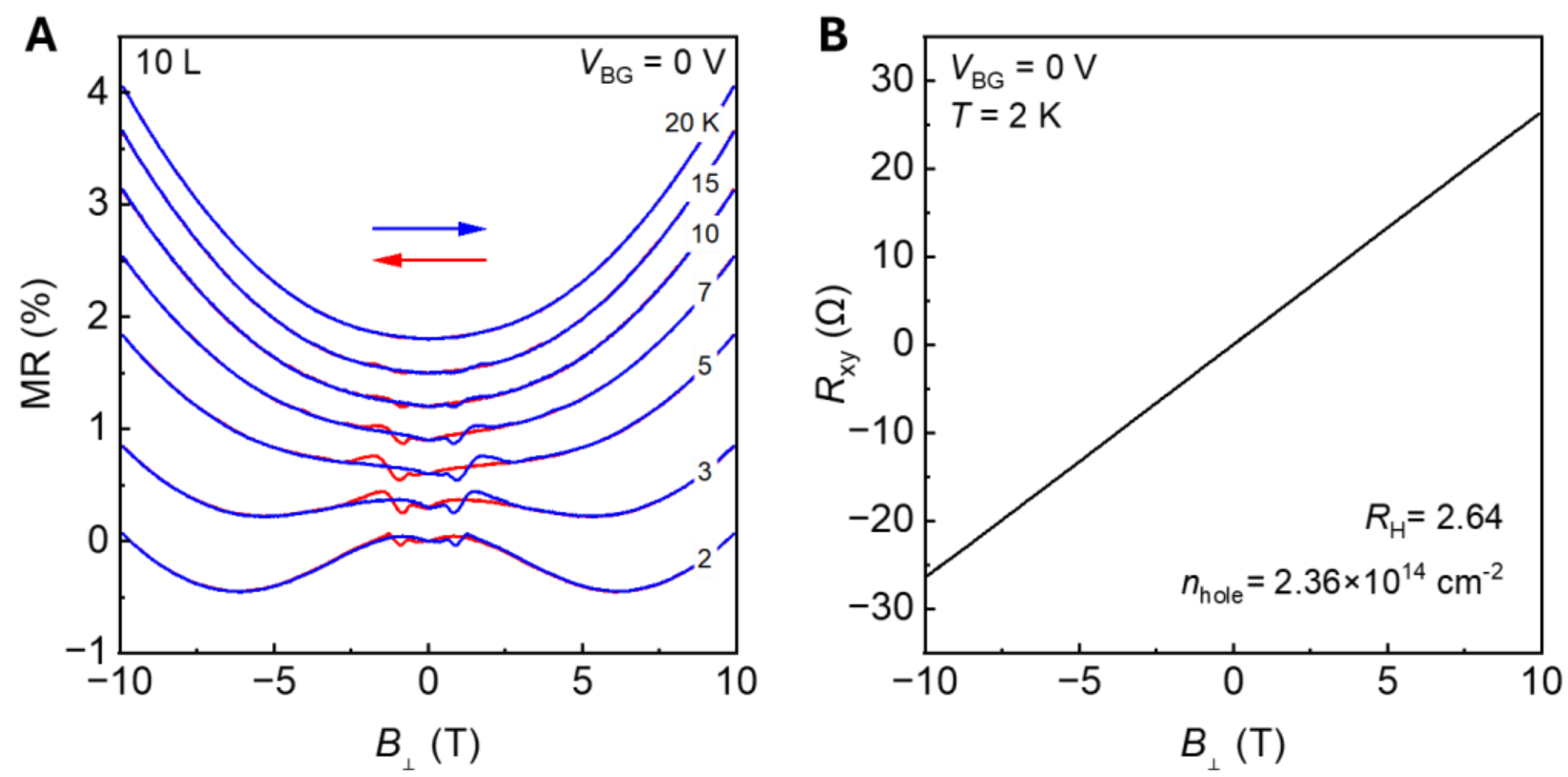

Fig. S6. Magnetotransport measurement of 10L Fe-doped $PtSe_2$ device. (**A**) Temperature-dependent MR ratio as a function of magnetic field at 0 back-gate voltage. (**B**) R_{xy} as a function of magnetic field at zero back-gate voltage and 2 K. R_H and n_{hole} represent 2D Hall coefficient and hole concentration, respectively.

Fig. S7A and B show the temperature-dependent MR ratio as a function of magnetic field of 7L and 8L devices, respectively. Similarly to 9 and 10L devices, both show hysteresis around zero field, indicating FM dominated magnetic behavior. However, the 7L device already starts to reveal very weak AFM behavior at higher magnetic field at 5 and 7K. This implies 7L is transitioning from FM to AFM dominated system as thickness decreasing, especially for 6L device, which is the main discussion in our work.

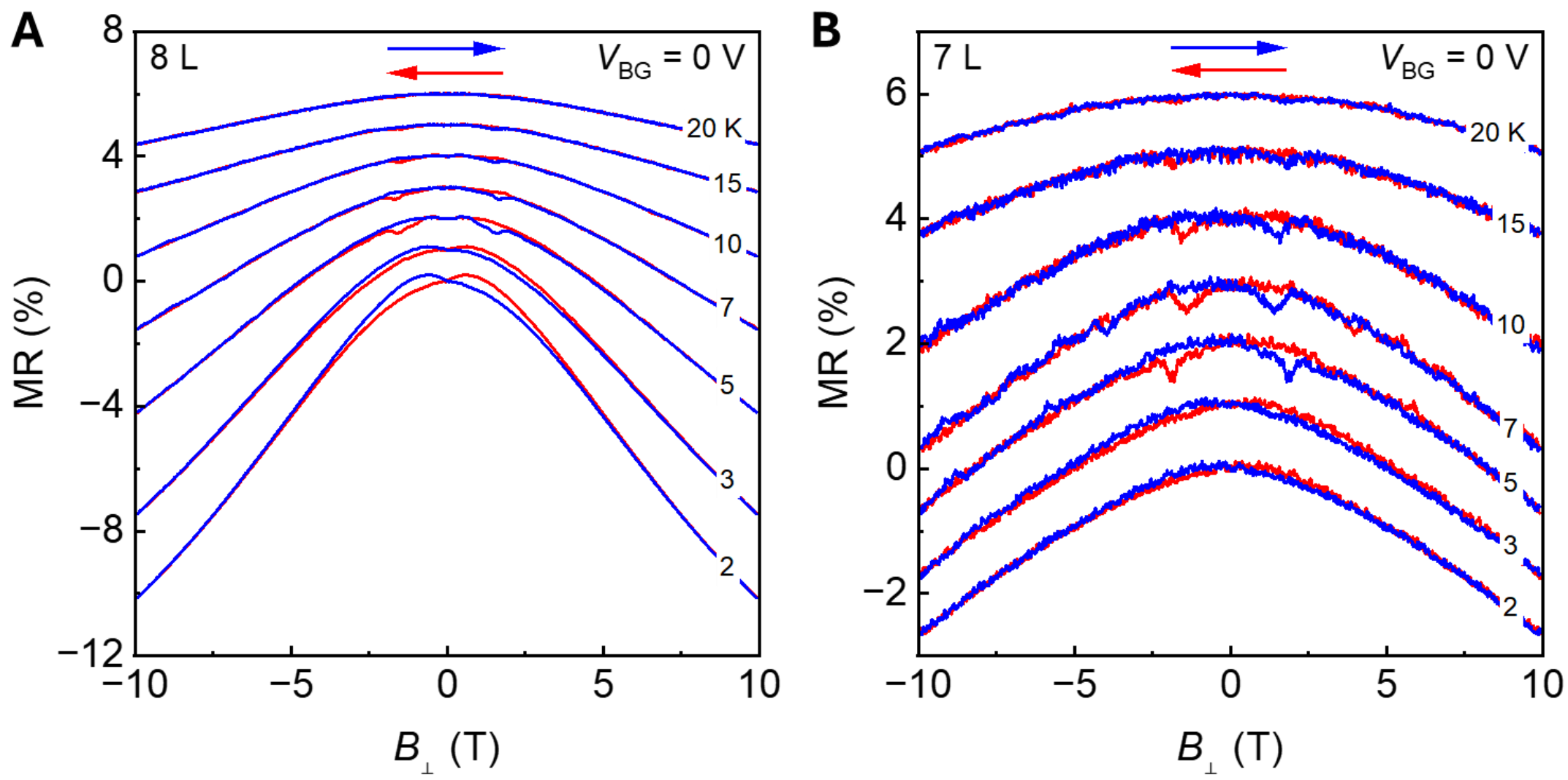

Fig. S7. Temperature-dependent MR ratio of 7L (A) and 8L (B) Fe-doped $PtSe_2$ device as a function of magnetic field at 0 back-gate voltage.

Additional measurements of the 6L device.

Fig. S8 shows the variation of the resistance with respect to the angle between the applied magnetic field and the Fe-doped $PtSe_2$ device, measured at 1.5 K, 0 V back-gate voltage, and 5 T of applied magnetic field. The angle between the applied magnetic field and the Fe-doped $PtSe_2$ device is shown schematically in the inset. For the angular dependence measurements, the device was rotated about the axis perpendicular to the applied current direction. The plotted data is normalized with respect to the resistance for the perpendicularly applied magnetic field. From the angular dependence measurement, the calculated magnetic anisotropy ($\frac{R_{\perp}-R_{\parallel}}{R_{\perp}}$) turns out to be 3%, suggesting a weak magnetic anisotropy.

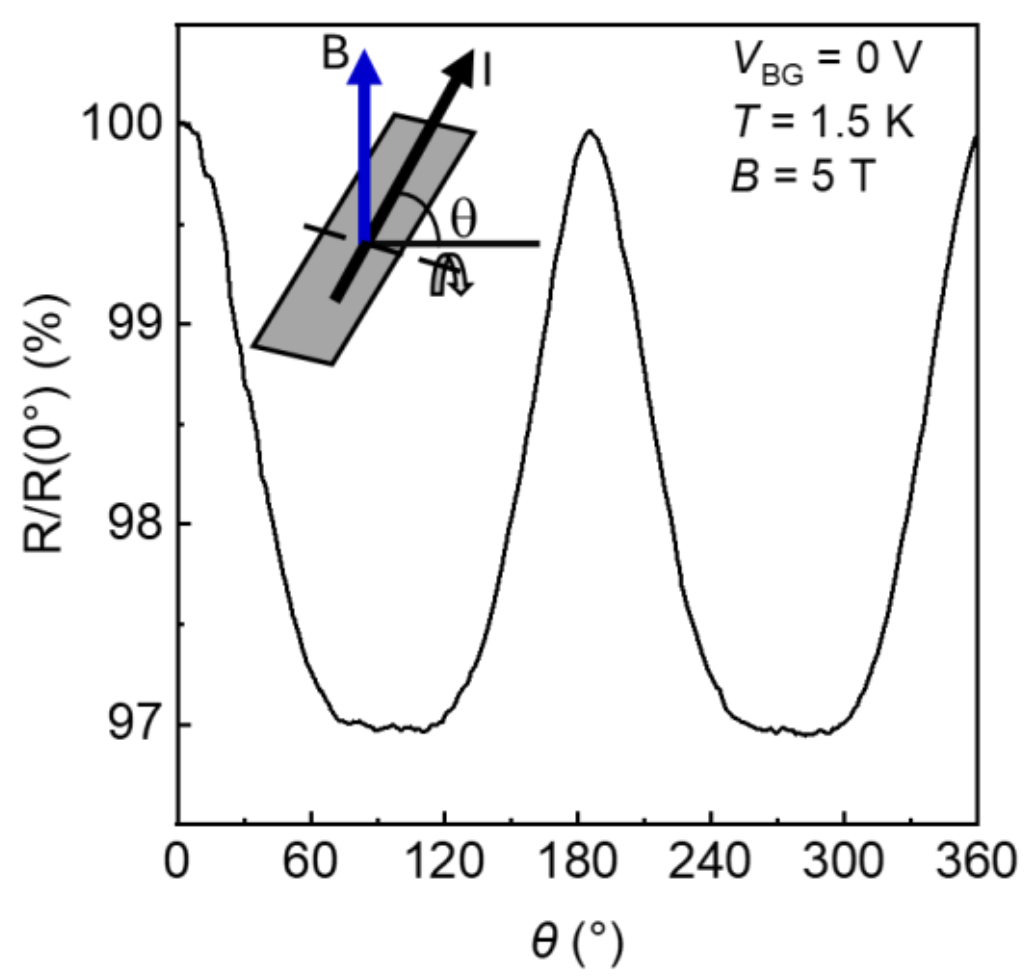

Fig. S8. Angular-dependent resistance measurement of 6L device. Measured at 1.5 K, the applied magnetic field of 5 T and zero back-gate voltage. The inset schematic shows the direction of the applied magnetic field with respect to the device.

For AFM materials, any kind of hysteresis in the R_{xy} could be seen only if the AFM order forms either non-collinear or non-coplanar non-trivial spin structure. In addition to any non-trivial AFM spin structure (if it is present), presence of finite momentum space Berry curvature also necessary to generate hysteresis in R_{xy}. The absence of any hysteresis in Fig. S9 suggests that the field-induced spin reorientation responsible for the MR hysteresis does not produce a sizable Berry curvature or anomalous Hall conductivity. This is consistent with a collinear or weakly canted antiferromagnetic state.

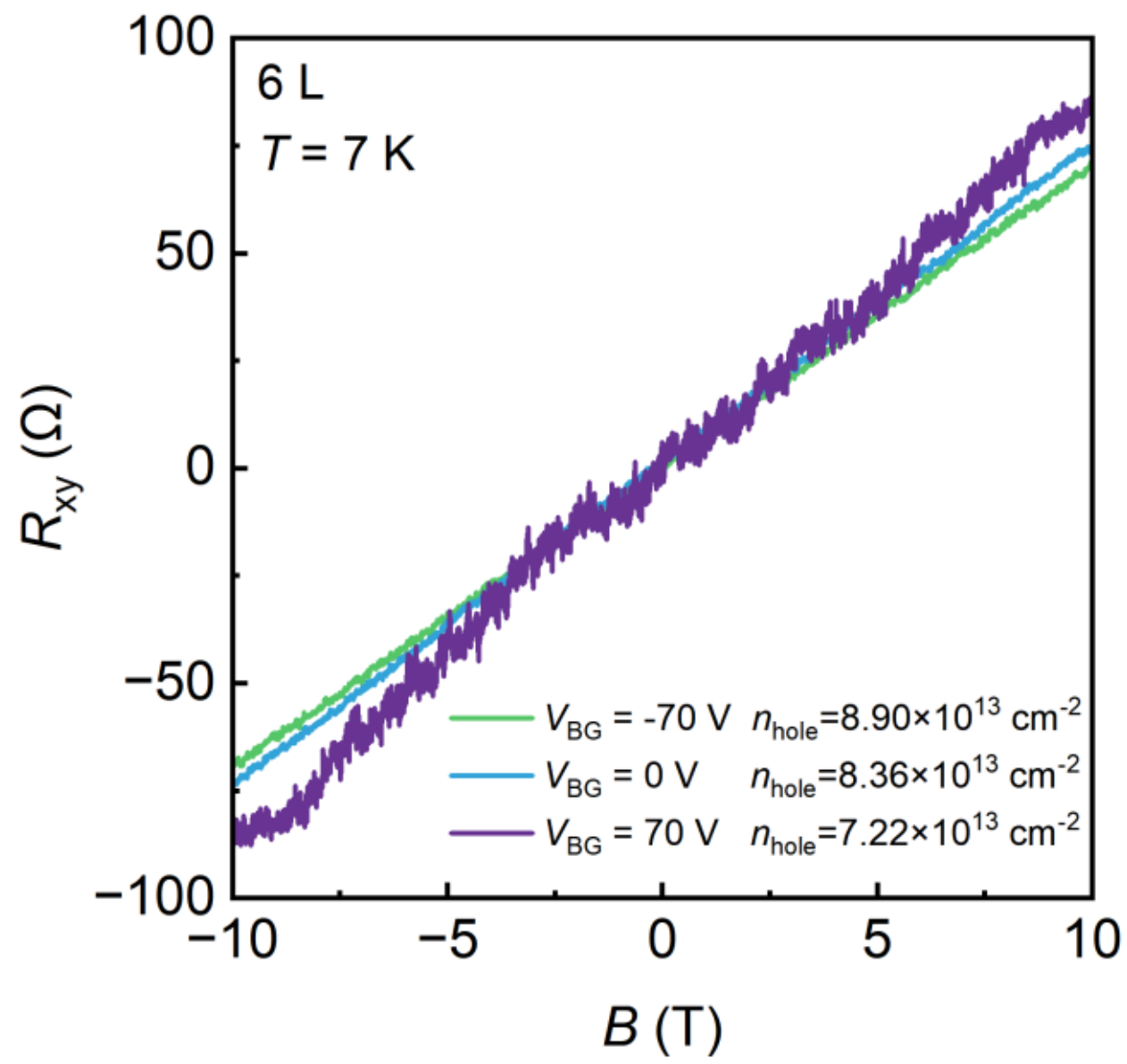

Fig. S9. Gate-dependent R_{xy} as a function of magnetic field of 6L device at 7 K. n_{hole} represents hole concentration.

Fig. S10A-C show the MR ratio as a function of magnetic field of 6L device at different temperatures for -70, 0, and 70 V back-gate voltages respectively (the data measured at different temperatures are plotted with vertical offsets for better visual clarity). As back-gate voltage increases from -70 V to 70 V, we observe the magnetic-field induced hysteresis with increased intensity, while the hysteresis at each back-gate voltage fades away as temperature increases. The temperatures where hysteresis disappears are approximately 25, 35, and 55 K, which are estimated as the corresponding Neel temperature for -70, 0 and 70V.

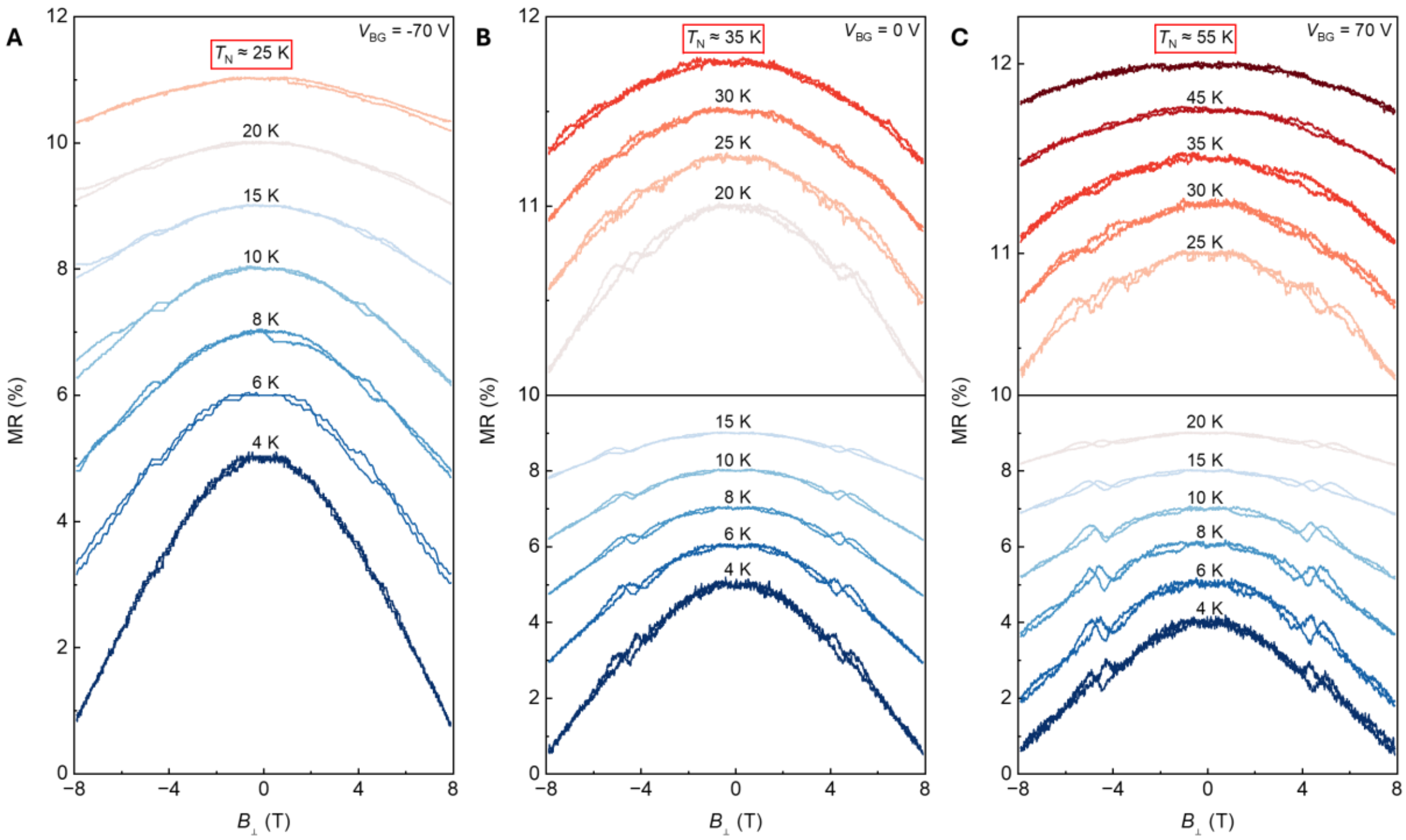

Fig. S8

Fig. S10. Temperature-dependent MR measurement of 6L device. Temperature-dependent MR ratio as a function of magnetic field of 6L device at different back-gate voltage: (**A**) (-70 V), (**B**) (0 V) and (**C**) (70 V). Different Y-axis scales are applied in Top and bottom panels in (B) and (C) to present the variation in MR ratio more clearly.

Dashed lines in Fig. S11A show the MR ratio as a function of magnetic field measured at 7 K and 0 V back-gate voltage, sweeping directions of the magnetic field are indicated by arrows. To get clearer details about the magnetic field-induced hysteresis, we tried to subtract the background of MR ratio by fitting it with a higher order polynomial function, which are represented by solid lines. After subtracting the background, the data is plotted in Fig. S11B. The strength of hysteresis is defined as ΔR ratio, as shown by the red double headed arrow. Similar background subtraction analysis has been done for all measured MR ratios in Fig. S10. The temperature dependence of the ΔR ratio at back-gate voltages of -70, 0, and 70 V are plotted in Fig. S11C. As the temperature increases, the ΔR ratio decreases to zero at 25, 35 and 55 K for -70, 0 and 70 V.

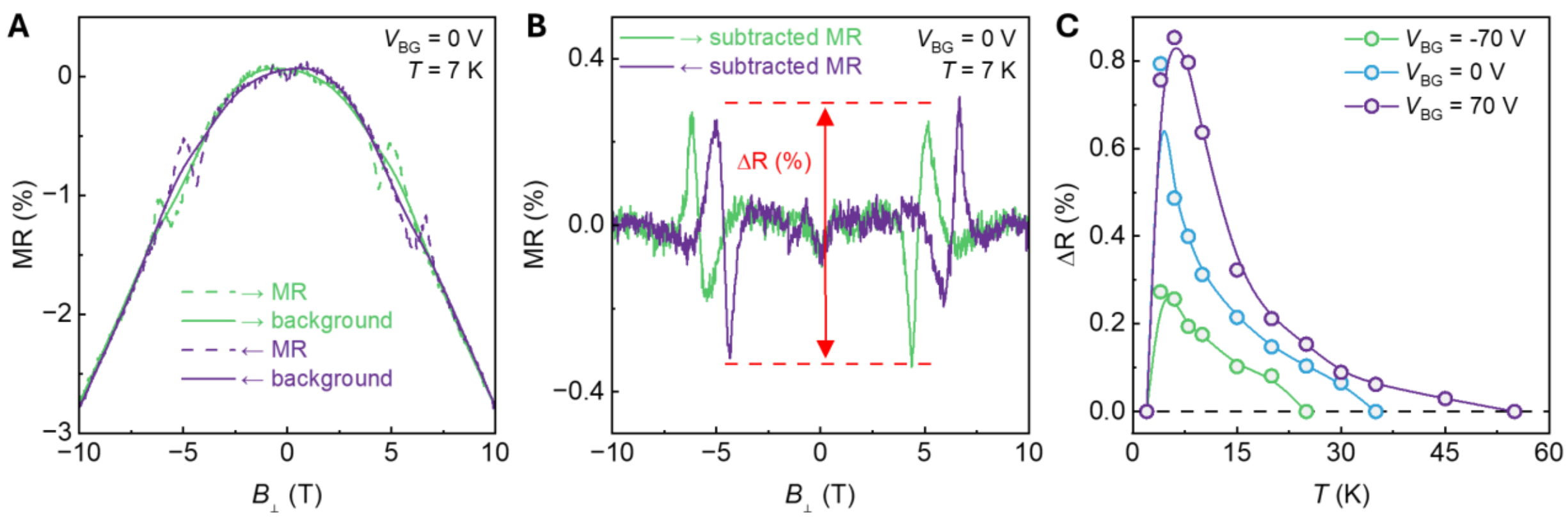

Fig. S11. Extraction of hysteresis intensity of 6L device. (**A, B**) The representative MR ratio as a function of magnetic field of 6L device before (A) and after (B) subtracting the background, which is defined as solid lines in a. The ΔR ratio is defined as red arrow bar in (B). (**C**) Temperature dependence of the ΔR ratios extracted from Fig. S10. at different back-gate voltages**.**

Fig. S12A shows the range of temperature at which hysteresis in MR is observed of 6L device for three different measured back-gate voltages. For -70, 0, and 70 V back-gate voltage hysteresis in MR is observed in the temperature range of 4-25 K, 4-35 K, and 4-55 K respectively. For all the three measured back-gate voltages hysteresis in MR start appears at 4 K, where magnetic ordering is strong, application of back-gate voltage could not completely switch off the field-induced change in the magnetic structure. However, at round 25 K, hysteresis in MR is present for 0 and 70 V, but absent for -70 V of back-gate voltage, which means 25 K and onwards by application of back-gate voltage the magnetic ordering can be completely turned off and turned on. To understand the temperature evolution of magnetic hysteresis, MR at different temperatures of the 6L device in the range of 1.5-7 K and at 0 V back-gate voltage are plotted in Fig. S12B. In the low-temperature regime 1.5-2 K, MR does not show any hysteresis. From 3 K onward hysteresis in MR started appearing and increases as the temperature increases. The hysteresis in MR which is caused by magnetic field-induced change in the spin structure can be understood from the classical Ising Hamiltonian (*H*) in an applied magnetic field:

$$H = -\frac{1}{2}J\sum_{i,j}\boldsymbol{S}_i^z.\boldsymbol{S}_j^z - \sum_i \boldsymbol{H}.\boldsymbol{S}_i^z$$

For the first antiferromagnetic (AFM) exchange interaction term summation is over the nearest neighbors, whereas the second term accounts for the field-induced Zeeman energy at finite magnetic field; $\boldsymbol{S}_i^z$ is the moment on the *i*th site with its own local axis z_i. The absence of hysteresis in the range of 1.5-2 K, and appearing from 3 K onward, suggests that in the low-temperature regime (1.5-2 K), strength of the AFM interaction is strong enough, therefore application of magnetic field (up to 10 T) could not make field-induced any modification of the spin structure. Whereas 3 K and above, due to finite thermal energy the AFM interaction strength decreases, therefore application of magnetic field causes field-induced modification in the spin structure and hysteresis in MR appears. On the other hand, in the higher temperature regime strength of the AFM becomes very weak, and does not cause any hysteresis in MR, but still, it shows negative MR. Therefore, we define Neel temperature of at which hysteresis in MR vanishes for different measured back-gate voltages.

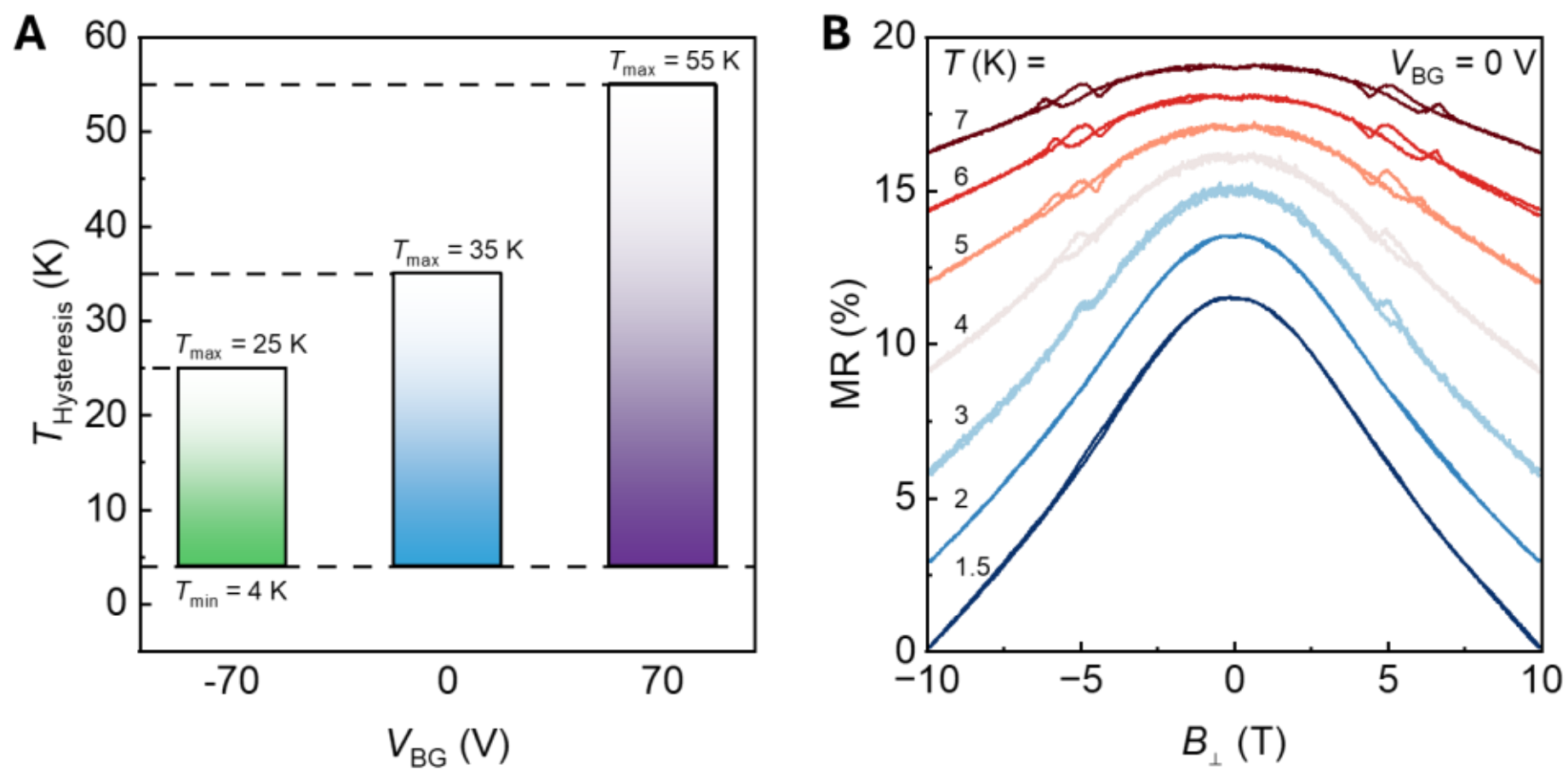

Fig. S12. Gate-tunable Neel temperature in 6L device. (**A**) Temperature range of 6L device in which hysteresis in MR is observed measured at different back-gate voltages. (**B**) Temperature-dependent MR ratio of 6L device as a function of magnetic field in the range of 1.5-7 K, and at zero back-gate voltage.

Fig. S13A shows the resistance as the function of temperature from 1.5 K to 300 K, measured at back-gate voltages of -70, 0, and 70 V, respectively. For all the three measured back-gate voltages, the resistance decreases monotonically as the temperature increases. However, a sharp variation in resistance is observed in the lower temperature regime. Earlier studies for intrinsic magnetic systems also show such behavior in the temperature-dependent resistivity measurement at their magnetic ordering temperatures. Therefore, the sudden change in the resistance measured in our 6L device could also be associated with the onset of the magnetic ordering in the crystal. To estimate the magnetic ordering temperature more accurately, we have plotted the first order derivative of resistance with respect to temperature (d*R*/d*T*) in Fig. S13B for all the three measured back-gate voltages. The derivative of resistance changes slowly within the higher temperature regime (100-300 K), but drastically below 100 K. We have extrapolated two straight lines for the high and low temperature regime as shown by dashed lines. The x-coordinates of intersection points of those two extrapolated straight lines at each back-gate voltages are assigned as their Neel temperature, which refer to 23, 39 and 58 K.

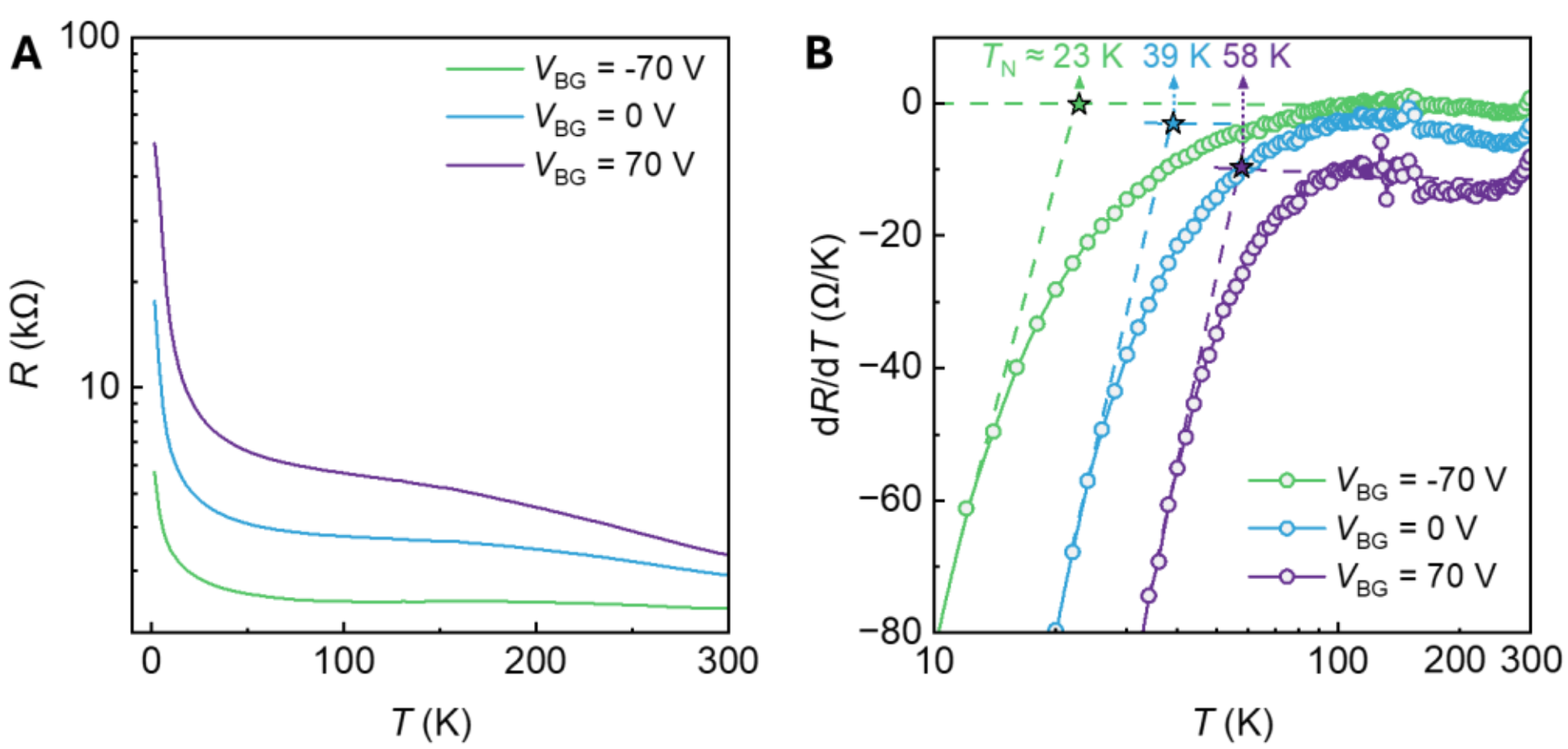

Fig. S13. Temperature-dependent resistance measurement of 6L device. (**A**) Resistance as a function of temperature of 6L device at different back-gate voltages. (**B**) The first derivative of curves in (A). Dashed lines and the corresponding x-coordinates of their intersection points indicate the extracted Neel temperature.

Additional measurements of the 5L device.

It is well established that the electronic structure of pristine $PtSe_2$ is strongly thickness dependent, evolving from a semiconducting state in the few-layer limit to a metallic state in crystals thicker than 5 layers. To explicitly rule out fabrication-induced effects, we performed control experiments on undoped $PtSe_2$ by fabricating devices based on pristine 5-layer $PtSe_2$ flakes using the same processing protocol as for the Fe-doped devices.

We characterized two pristine 5-layer $PtSe_2$ devices: one employing a conventional SiO_2 back-gate and the other using an ionic-liquid top gate. As shown in Fig. S14, both devices exhibit clear gate-tunable transport with sizeable off state, consistent with the small-bandgap electronic structure of few-layer $PtSe_2$. As expected, the ionic-liquid-gated device shows stronger tunability due to its higher gating efficiency, while the SiO_2-gated device displays qualitatively similar behavior.

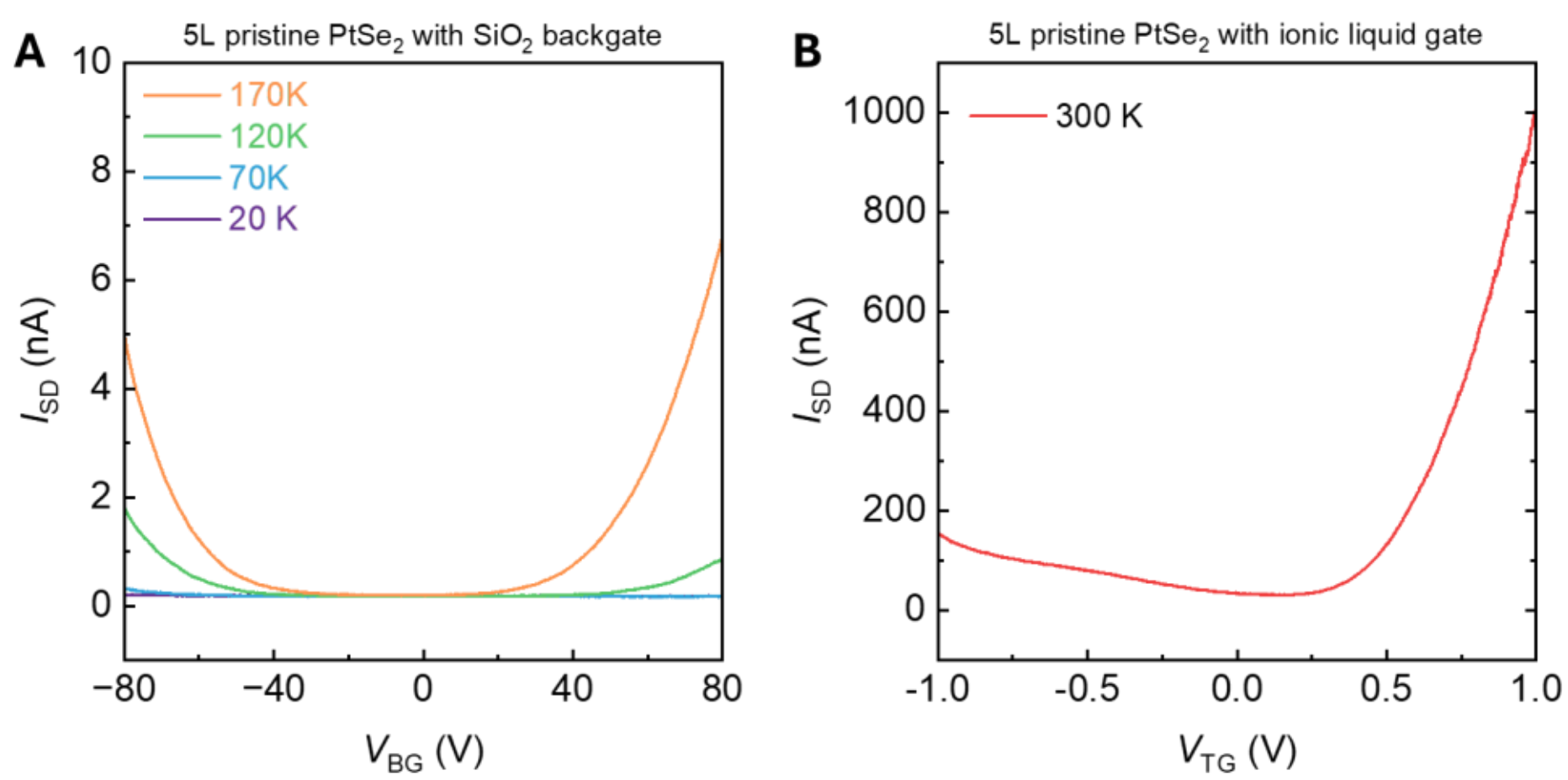

Fig. S14. Transfer characteristics of two pristine 5L Fe doped $PtSe_2$ devices with conventional SiO_2 back-gate (A) and ionic-liquid top gate (B).

Fig. S15 shows the MR ratio as a function of magnetic field of 5L device at back-gate voltage of -60 V and temperature in the range of 25-70 K (the data measured at different temperatures are plotted with vertical offsets for better visual clarity). The MR ratio also shows magnetic field-induced finite hysteresis as 6L device. With increasing temperature, the strength of the hysteresis decreases, and vanishes around 70 K.

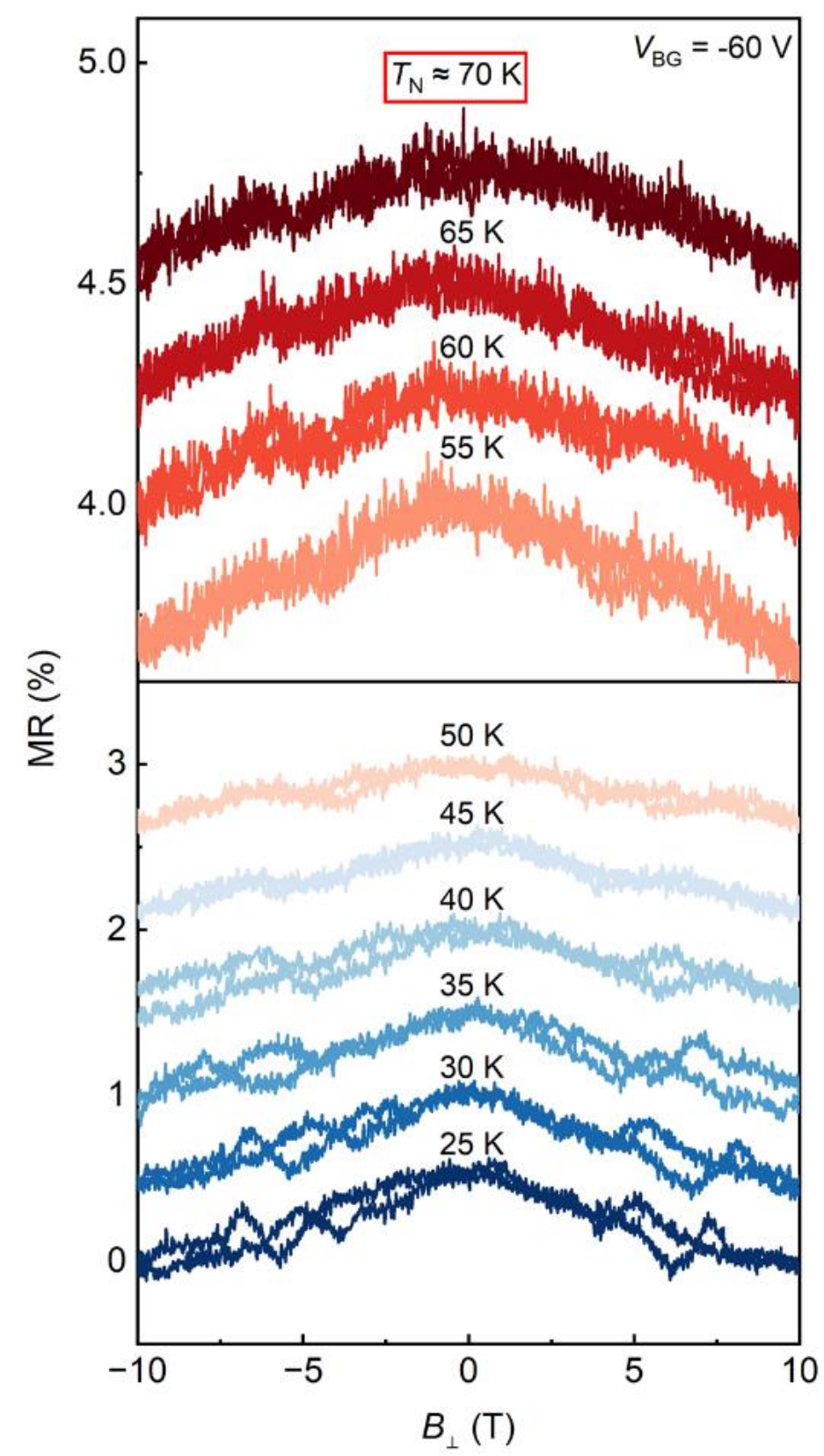

Fig. S15. Temperature-dependent MR measurement of 5L device. Temperature-dependent MR ratio as a function of magnetic field of 5L device at back-gate voltage of -60 V. Different Y-axis scales are applied in Top and bottom panels to present the variation in MR ratio more clearly.

Fig. S16A shows the resistance as the function of temperature from 20 K to 300 K, measured at back-gate voltages of -80, -60 and -40 V, respectively. The corresponding first order derivative of resistance with respect to temperature (d*R*/d*T*) for all three measured back-gate voltages are plotted in Fig. S16B, and their Neel temperatures are estimated to be 52, 69 and 82 K, by using the same method as 6L device.

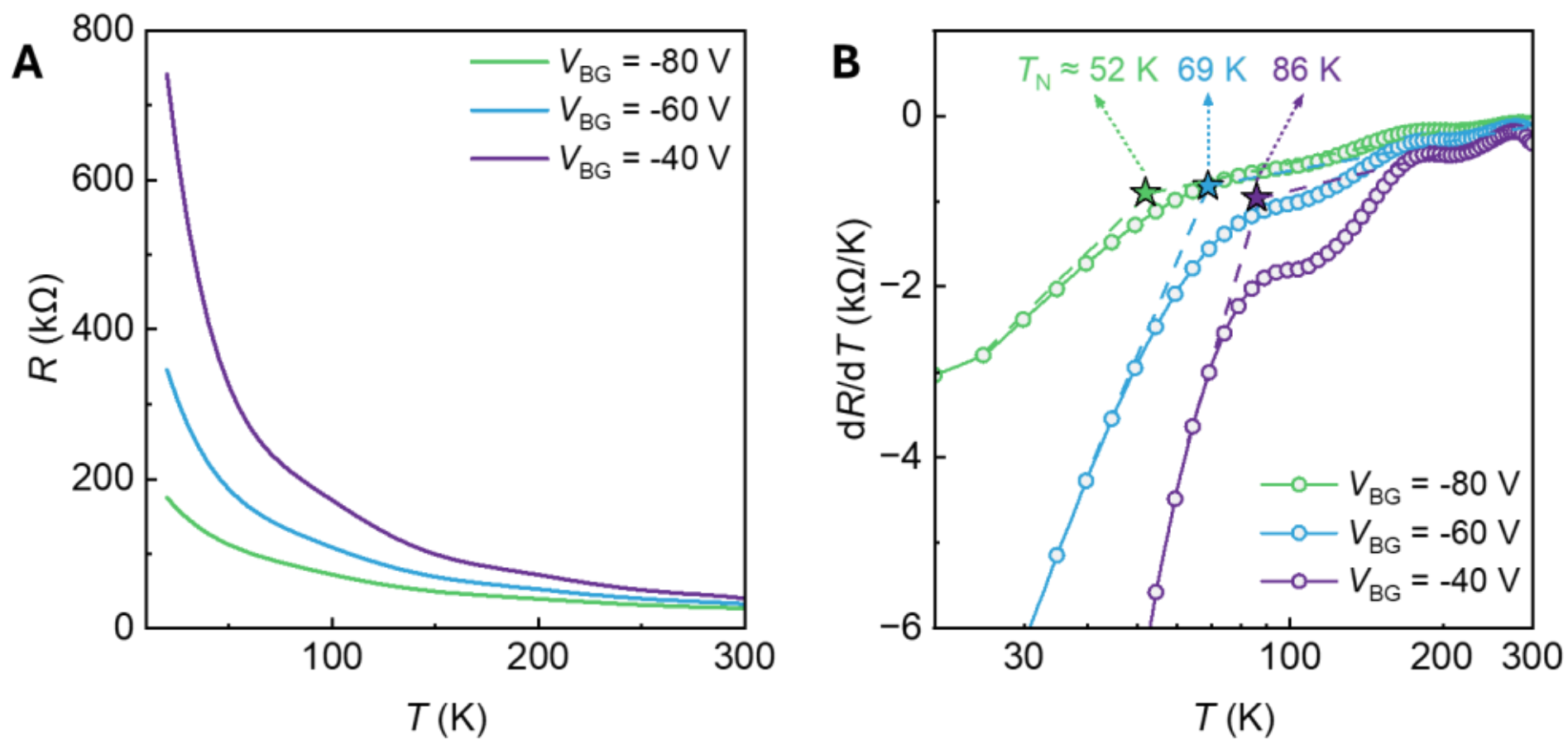

Fig. S16. Temperature-dependent resistance measurement of 5L device. (**A**) Resistance as a function of temperature of 5L device at back-gate voltage of -60 V. (**B**) The first derivative of curves in (A). Dashed lines and the corresponding x-coordinates of their intersection points indicate the extracted Neel temperature.

Additional computational analysis.

The role of the on-site Coulomb interaction on the Fe d-states is investigated by performing DFT+U calculations for U values up to 4 eV (Fig. S17A). In this range, the impact on the low-energy band structure and on the magnetic moments is negligible: the overall band topology and spin polarization near the Fermi level remain essentially unchanged. Considering that the equivalent doping concentration in 3×3 supercell is 11.1%, which is higher than the ~5.5% confirmed by our EDX and XPS results, we perform additional calculations (Fig. S17B, C) for 4×4 (6.25% Fe) and 5×5 (4% Fe) supercells, indicating the main qualitative features, such as the Fe-atom magnetization, are robust against this increase in supercell size.

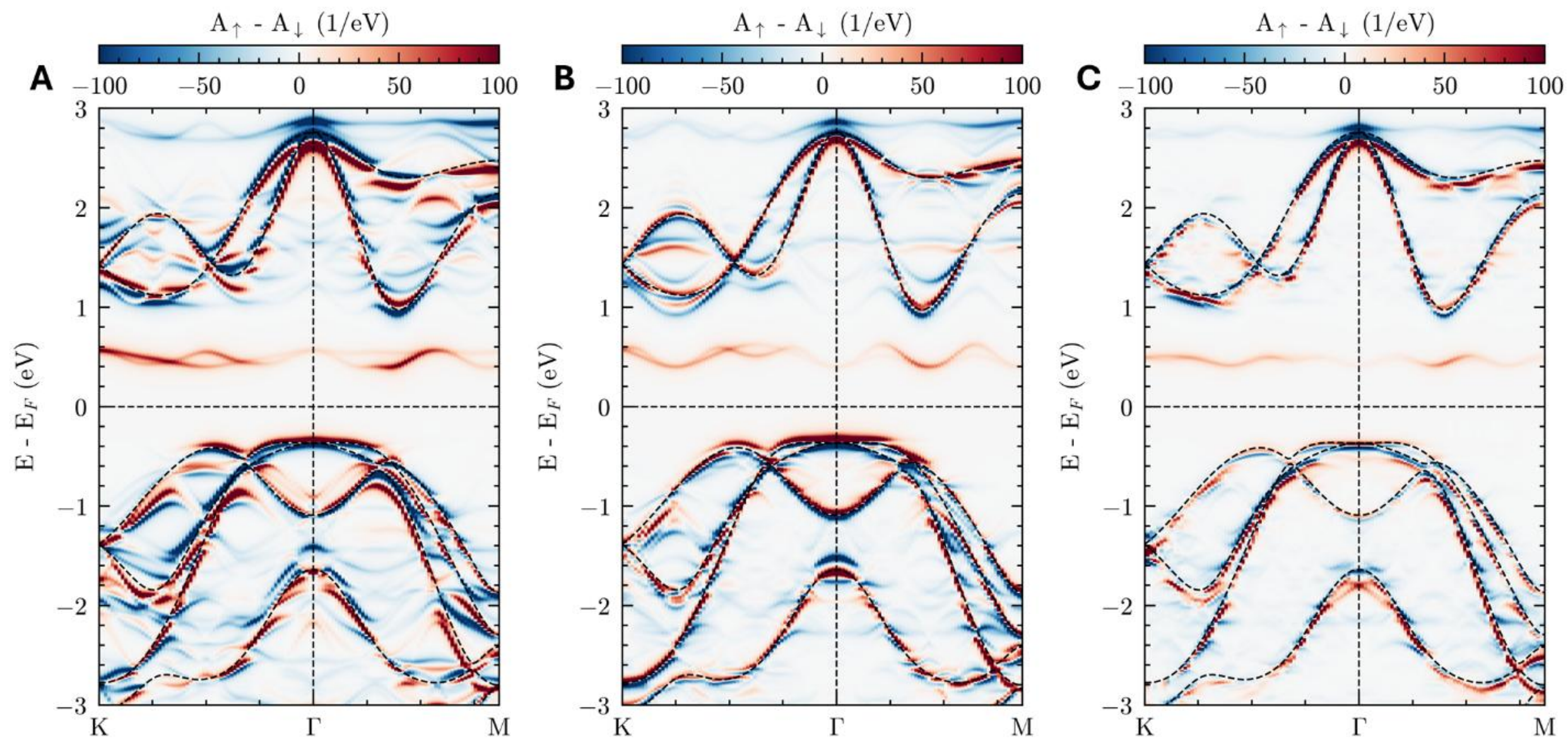

Fig. S17. Supercell size dependence of unfolded band structure. Unfolded band structures for (**A**) 3×3, (**B**) 4×4, and (**C**) 5×5 single-Fe-doped $PtSe_2$ monolayer supercells. All calculations are performed with a Hubbard U = 4 eV applied to the Fe d-states. The color code indicates spin polarization, with the dashed line representing the pristine $PtSe_2$ monolayer band structure.

In addition to the monolayer, we perform bilayer DFT calculations with Fe doping in the top layer (Fig. S18A) and bulk DFT calculations for a 3x3x3 supercell with a single Fe atom (Fig. S18B). In both cases, DFT strongly underestimates the band gaps; however, for the monolayer, this problem is almost negligible because the DFT band gap is sufficiently large. We observe strong n-type doping from Fe impurities for the bilayer and bulk cases. However, the spin-resolved band structure, aside from band gap renormalization, shows qualitatively the same spin-splitting modifications as the monolayer case.

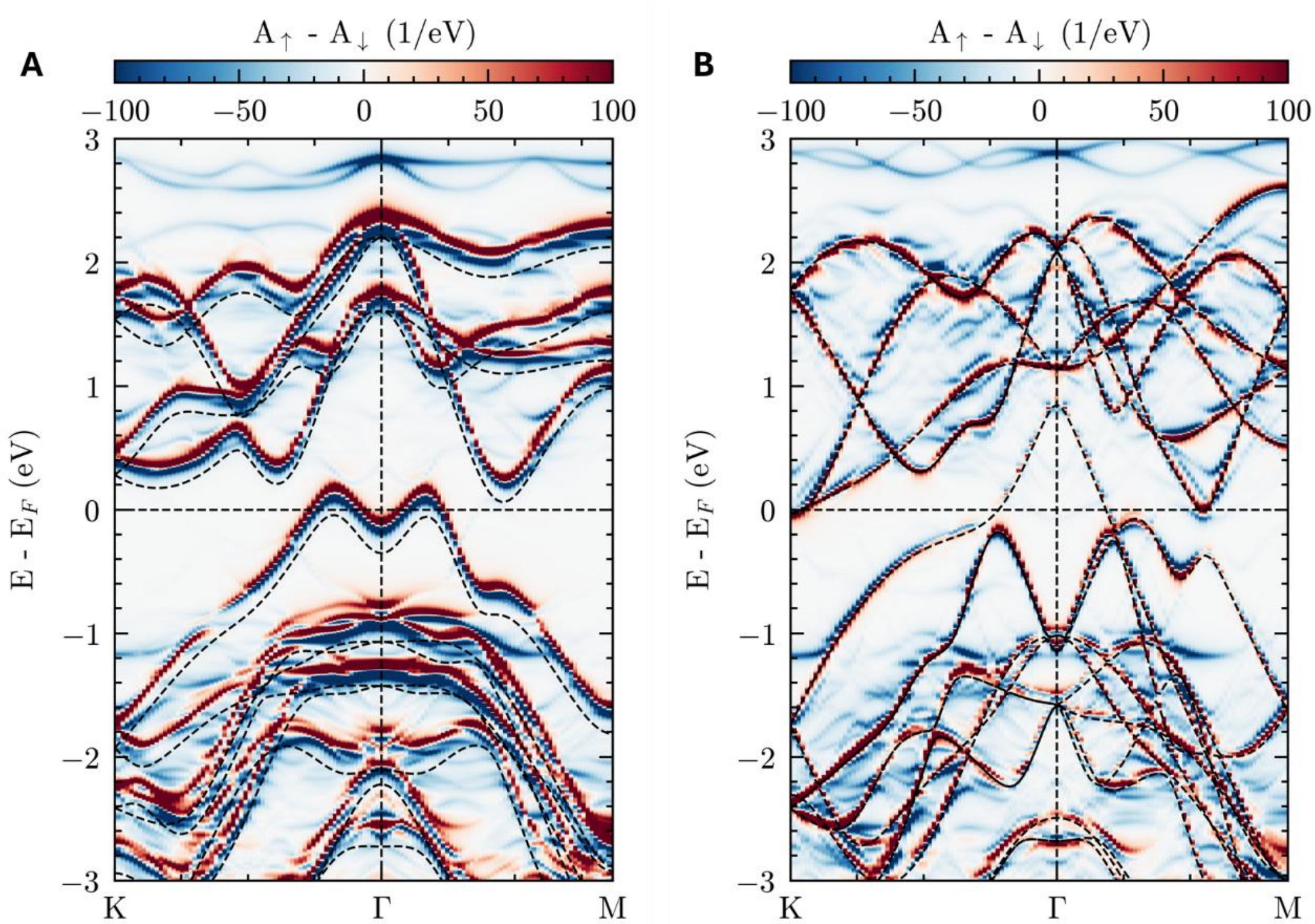

Fig. S18. Unfolded band structure for 3x3 single-Fe-doped $PtSe_2$ bilayer supercell (A) and 3x3x3 single-Fe-doped $PtSe_2$ bulk supercell (B). Calculations are performed with a Hubbard U of 4 eV applied to the Fe d-states. The color code indicates spin polarization, with the dashed line representing the pristine $PtSe_2$ band structure.

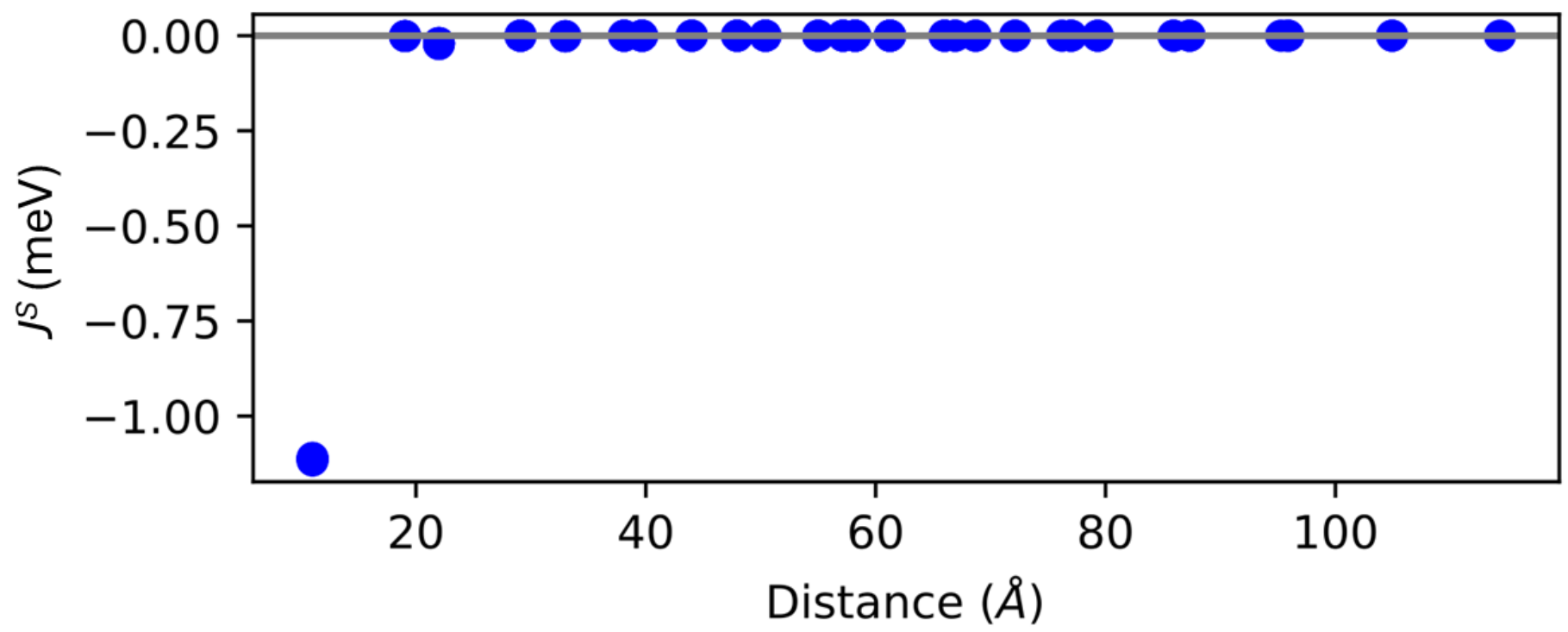

Fig. S19. Distance dependence of the Fe-Fe superexchange interaction. Fe-Fe superexchange interaction as a function of separation for a 3×3 $PtSe_2$ monolayer supercell with a single Fe dopant, calculated without a Hubbard U and for an electron doping of -1 electron per Fe atom. Contributions from next-nearest and more distant neighbors are negligible.

Calculations are performed for the 3×3 supercell because for larger supercells the exchange interaction is smaller than 0.1 meV and the Fe atom in the 4×4 and larger supercells can be regarded as an isolated magnetic moment.

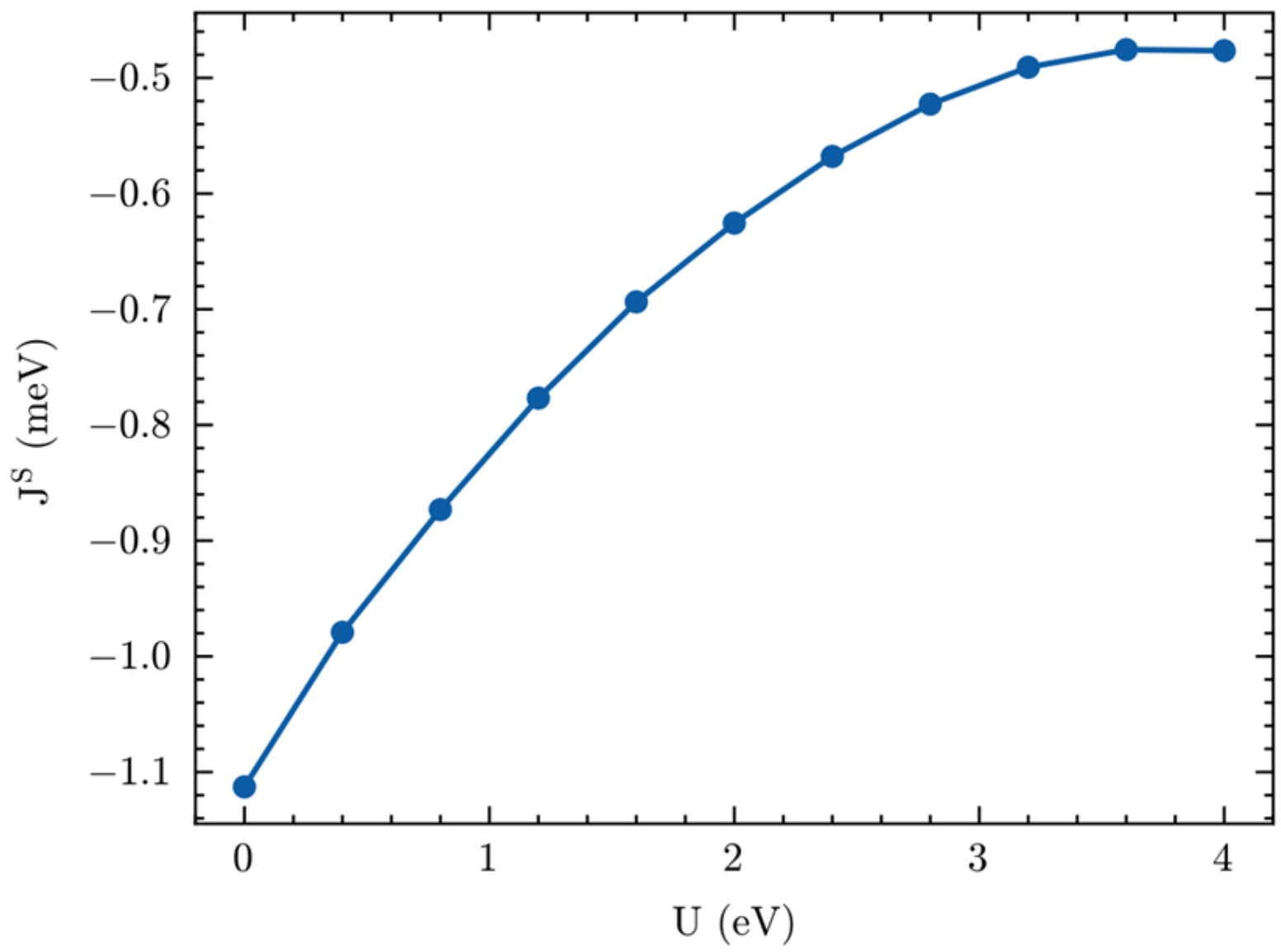

Fig. S20. Hubbard U dependence of the Fe-Fe superexchange parameter. Fe-Fe superexchange coupling as a function of the Hubbard U applied to the Fe d-states for the 3×3 single-Fe-doped $PtSe_2$ monolayer supercell in the semiconducting regime.

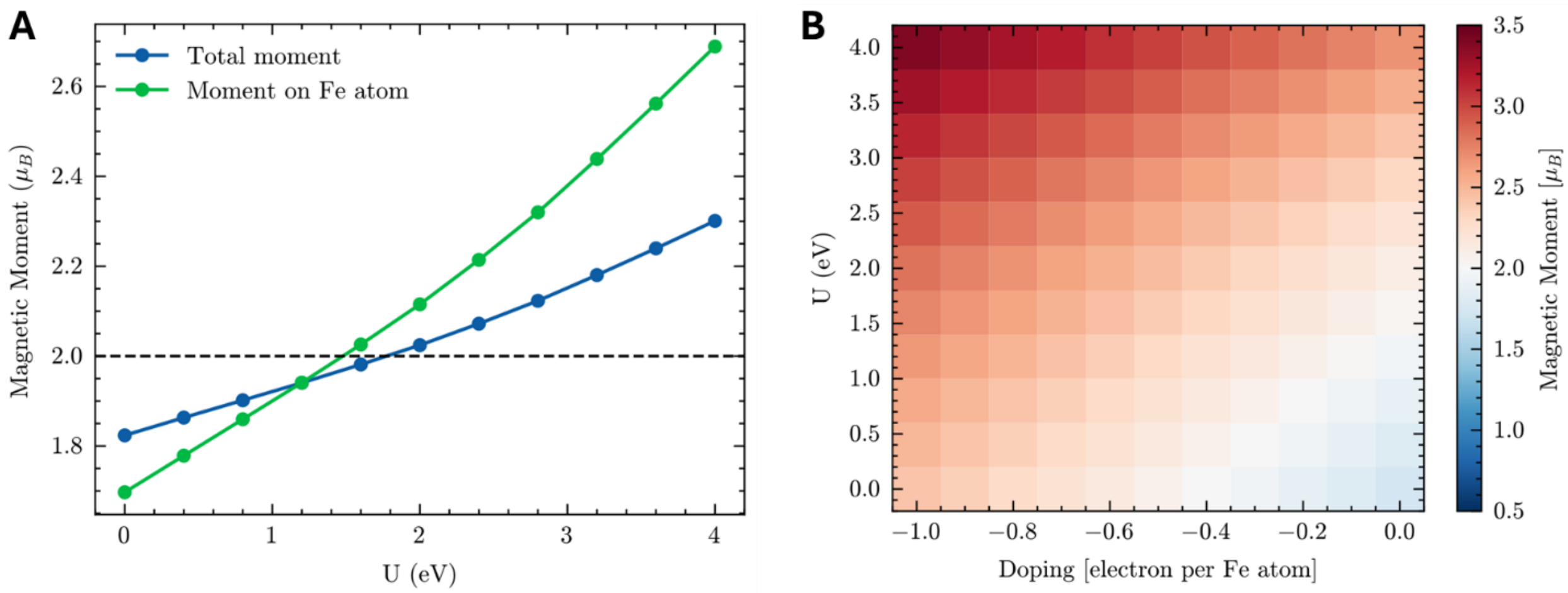

Fig. S21. Hubbard U and doping dependence of the Fe moment. (**A**) Magnetic moment of Fe as a function of the Hubbard U applied to the Fe d-states for the 3×3 single-Fe-doped $PtSe_2$ monolayer supercell (solid green line). The Hubbard U only quantitatively renormalizes the local Fe magnetic moment. The solid blue line corresponds to the total local magnetic moment (sum over all atoms), while the dashed line corresponds to the magnetization integrated over the supercell. (**B**) Magnetic moment of Fe as a function of the Hubbard U and doping applied to the Fe d-states for the 3×3 single-Fe-doped $PtSe_2$ monolayer supercell.
